# A map of protein dynamics during cell-cycle progression and cell-cycle exit

**DOI:** 10.1371/journal.pbio.2003268

**Published:** 2017-09-11

**Authors:** Sara Gookin, Mingwei Min, Harsha Phadke, Mingyu Chung, Justin Moser, Iain Miller, Dylan Carter, Sabrina L. Spencer

**Affiliations:** 1 Department of Chemistry and Biochemistry, University of Colorado-Boulder, Boulder, Colorado, United States of America; 2 Department of Electrical, Computer & Energy Engineering, University of Colorado-Boulder, Boulder, Colorado, United States of America; 3 Department of Chemical and Systems Biology, Stanford University School of Medicine, Stanford, California, United States of America; Gurdon Institute, University of Cambridge, United Kingdom of Great Britain and Northern Ireland

## Abstract

The cell-cycle field has identified the core regulators that drive the cell cycle, but we do not have a clear map of the dynamics of these regulators during cell-cycle progression versus cell-cycle exit. Here we use single-cell time-lapse microscopy of Cyclin-Dependent Kinase 2 (CDK2) activity followed by endpoint immunofluorescence and computational cell synchronization to determine the temporal dynamics of key cell-cycle proteins in asynchronously cycling human cells. We identify several unexpected patterns for core cell-cycle proteins in actively proliferating (CDK2-increasing) versus spontaneously quiescent (CDK2-low) cells, including Cyclin D1, the levels of which we find to be higher in spontaneously quiescent versus proliferating cells. We also identify proteins with concentrations that steadily increase or decrease the longer cells are in quiescence, suggesting the existence of a continuum of quiescence depths. Our single-cell measurements thus provide a rich resource for the field by characterizing protein dynamics during proliferation versus quiescence.

## Author summary

The cell cycle is by nature highly dynamic, but we lack a standardized map of how core cell-cycle regulators change over time. In this study, we used time-lapse microscopy to track the dynamics of key cell-cycle proteins in individual human cells and found several unexpected patterns, even for well-studied proteins such as Cyclin D1. Our data provide a rich resource for those focused on the cell cycle, or on any biological process that is impacted by the cell cycle, by providing a series of maps of protein dynamics during cell-cycle progression and cell-cycle exit.

## Introduction

Cellular proliferation is driven by the mitotic cell cycle, a highly regulated process consisting of DNA synthesis (S phase) and mitosis (M phase), separated by gap phases (G1 and G2). Decades of cell-cycle research have led to in-depth understanding of the biochemical processes involved in cell-cycle progression, but the temporal dynamics of these processes, and how they differ in non-cycling cells, are less well characterized. Simplified diagrams of cell-cycle dynamics are sometimes depicted in textbooks [1,2,3], but these diagrams are not always in agreement, typically only Cyclin dynamics are represented, and information on protein behavior during quiescence is absent. Thus, although the cell cycle is one of the most dynamic processes in biology, we lack quantitative information about the chronology of key events during cell-cycle progression versus cell-cycle exit.

**Fig 1:**
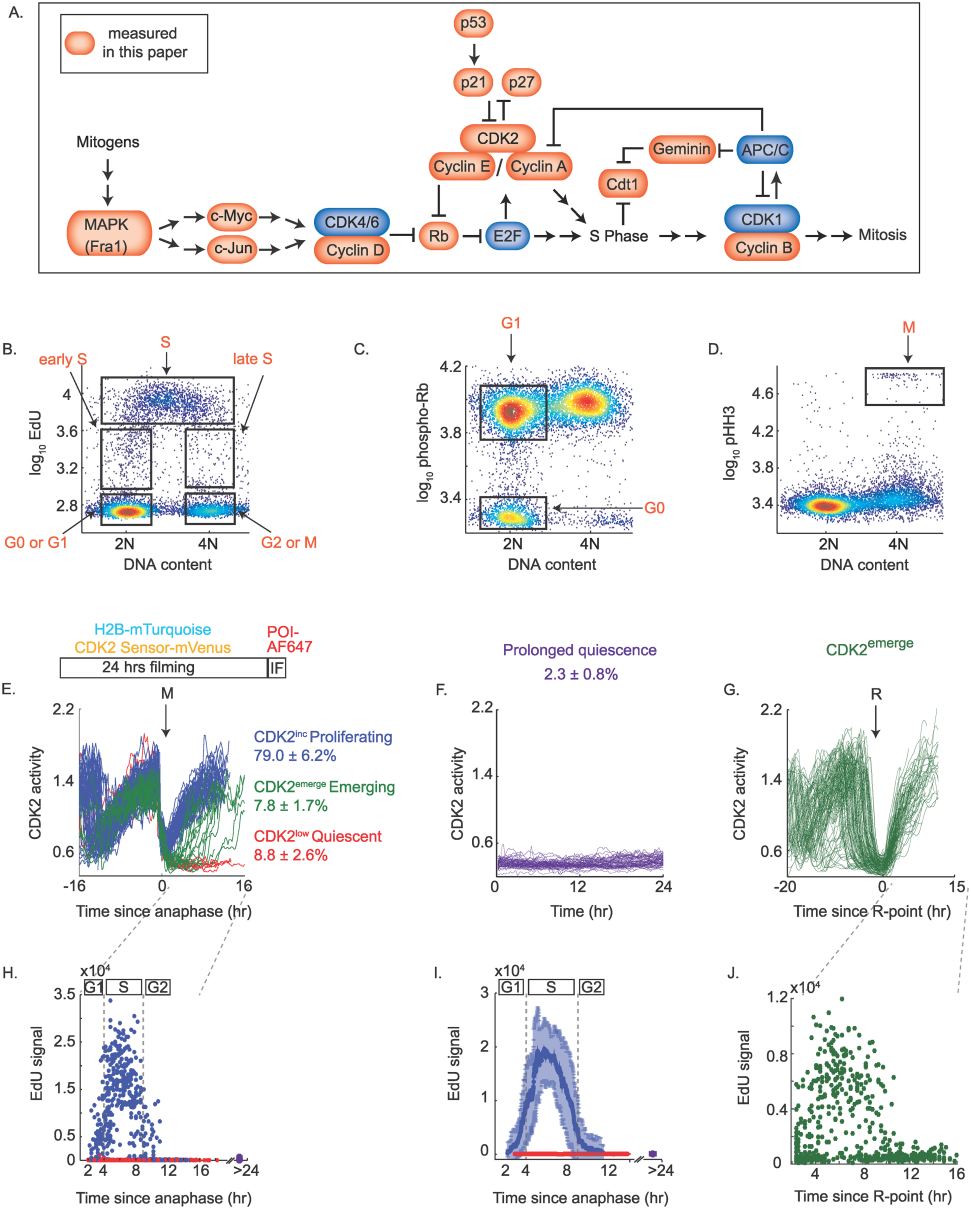
Single-cell IF methods for identifying cells in G0, G1, S, G2, and M phases of the cell cycle. (A) Cell-cycle signaling network depicting in red the proteins measured in this study. (B) Density scatter plot of EdU versus DNA content used to define G0/G1, early S, S, late S, and G2/M populations, as marked by the boxed populations. Note that y-axis units for Fig 1 B–D are in log base 10, such that, for example, a y-axis value of 2.8 = 10^2.8^ = 631; y-axis units are arbitrary fluorescence units. (C) Density scatter plot of phospho-Rb-S807/811 versus DNA content. EdU-negative cells with approximately 2N DNA content can be subdivided into a G0/quiescent population with hypo-phosphorylated Rb and a G1 population with hyper-phosphorylated Rb, as marked by the boxed populations. (D) Density scatter plot of phospho-Histone H3 versus DNA content. EdU-negative cells with approximately 4N DNA content can be subdivided into a G2 population with no phospho-Histone H3 signal and a mitotic population that is phospho-Histone H3-positive, as marked by the boxed population. (E) Top: Cells were tracked by live-cell imaging for 24 hours using H2B-mTurquoise as a nuclear marker, while simultaneously monitoring CDK2 activity using DHB-mVenus. Cells were then fixed and stained with various antibodies against proteins of interest (POI). The IF image was precisely aligned to the time-lapse movie using a custom jitter correction algorithm so as to match each cell’s history with its IF signal; see Materials and methods. Bottom: Single-cell traces of CDK2 activity aligned computationally to the time of the last anaphase of the movie; see Materials and methods. Traces were computationally classified and manually verified as CDK2^inc^ (blue), CDK2^low^ (red), or CDK2^emerge^ (green) based on CDK2 activity after mitosis: CDK2^inc^ traces must remain ≥0.5 for all frames post-anaphase; CDK2^low^ traces must remain <0.5 or all frames post-anaphase; CDK2^emerge^ traces initially enter the CDK2^low^ state and then emerge–these traces must remain <0.5 for at least 3 hours post-anaphase before rising at some point during the imaging period. The percentage of the total population in each category is indicated; error represents the standard deviation across 96 replicate wells. (F) Prolonged quiescent cells (purple) are defined as cells with CDK2 activity <0.6 for all 24 hours of imaging. These cells did not undergo mitosis. The percentage of the total population with this behavior is indicated; error represents the standard deviation across 96 replicate wells. (G) Single-cell traces of CDK2 activity for CDK2^emerge^ cells are aligned computationally to the time of CDK2 activity buildup (the R-point; see Materials and methods). Heterogeneity in the time these cells spent in quiescence prior to emerging can be seen as variability in the timing of the drop in CDK2 activity that marks the previous mitosis. (H) Time-lapse imaging of CDK2 activity in asynchronous cells was followed by a 15-minute pulse of 10 μM EdU, fixation, and visualization of the EdU signal in the Cy5 channel. The Cy5 fluorescence image was precisely aligned to the time-lapse movie using a custom jitter correction algorithm. This enabled matching of the CDK2 activity trace of each cell to its EdU level at the end of the movie. EdU levels were then reconstructed as a function of time-since-anaphase for CDK2^inc^ cells (blue dots) and CDK2^low^ cells (red dots). Each dot represents a single cell. The level of the EdU signal in prolonged quiescent cells is plotted at the 24-hour mark (purple dots). Based on these data, we designate G1, S, and G2 phases of the cell cycle using bars above this plot and apply this classification to Figs 3 and 5. Number of cells plotted: 978. (I) Moving average through the blue, red, or purple points from (H). Error bars represent standard deviation. (J) EdU levels as a function of time since CDK2 activity buildup (R-point). Number of cells plotted: 174. All data are from asynchronous MCF10A cells. Abbreviations: APC/C, anaphase-promoting complex/cyclosome; CDK2, Cyclin-Dependent Kinase 2; IF, immunofluorescence; POI, proteins of interest; R-point, Restriction Point; Rb, retinoblastoma protein.

An abbreviated explanation of the events surrounding cell-cycle entry and cell-cycle progression follows, with Fig 1A serving as a simplified network diagram. In quiescent or resting cells, Cyclin-Dependent Kinase (CDK) activities are low or off, and the master regulator of cell-cycle entry, the retinoblastoma protein (Rb), is in a non-phosphorylated state in which it binds and inhibits the E2F transcription factor. Cell-cycle entry can be triggered when resting cells receive extracellular mitogenic signals. Mitogenic signaling leads to Erk-dependent activation of transcription factors, such as c-Myc [4] and Ets-1 [5], which in turn up-regulate Cyclin D. Cyclin D binds its cognate Cyclin-Dependent Kinases, CDK4 and CDK6, which initiate hypo-phosphorylation of Rb. In the textbook model, this initial hypo-phosphorylation of Rb liberates the E2F transcription factor, a key driver of genes involved in the G1/S transition, including Cyclin E [6,7]. Transcriptional up-regulation of Cyclin E drives Cyclin-Dependent Kinase 2 (CDK2)/Cyclin E activity, leading to “hyper” phosphorylation of all 14 sites on Rb, and liberating additional E2F in a positive feedback loop. However, this model was recently called into question by the observation that E2F target genes were only up-regulated at the time of Rb hyper-phosphorylation and not with the initial hypo-phosphorylation [8]. Nevertheless, it is generally accepted that Rb hyper-phosphorylation marks passage through the Restriction Point (R-point) [9], defined as the time at which cells no longer require mitogens to complete the rest of the cell cycle [10]. Concordantly, activation of CDK2 was shown via single-cell time-lapse microscopy to mark cells that had passed the R-point [11].

At the beginning of S phase, Cyclin A protein levels begin to rise, and Cyclin A/CDK2 becomes the dominant source of CDK activity driving cells through S phase. DNA replication is initiated when origins of replication, previously prepared for replication by licensing factors such as Cdt1, fire due to phosphorylation by Dbf4-dependent kinase and CDK activities [12]. To prevent relicensing and re-replication of DNA, Cdt1 is degraded at the start of S phase by the E3 ubiquitin ligases SCF^Skp2^ and CRL4^Cdt2^ [13]. Any residual Cdt1 is bound and inhibited by Geminin, the levels of which rise during S and G2 [14,15]. Toward the end of S phase, Cyclin B levels rise rapidly, giving rise to Cyclin B/CDK1 activity that propels cells into mitosis [16]. The anaphase-promoting complex/cyclosome (APC/C) triggers exit from mitosis and is responsible for resetting the cell cycle at the end of mitosis via the degradation of Cyclin A, Cyclin B, Geminin, and many other substrates [17]. Cell-cycle progression is also controlled by protein inhibitors of CDKs, including p21 and p27, the ubiquitination and degradation of which promote S phase entry [18,19,20,21,22].

Cells can also temporarily exit the cell cycle by transitioning to a resting state, termed quiescence or G0. Relative to our knowledge of G1, S, G2, and M, the G0 phase remains poorly understood, both in terms of when and how cells transition into and out of G0 and in terms of a molecular definition of G0. Although there are multiple forms of quiescence, a universal feature of quiescence is lack of progression through the cell cycle [23]. Previous efforts to characterize quiescence in human cells have used serum starvation, contact inhibition, or loss of adhesion to induce quiescence, identifying a set of genes expressed across all three modes of quiescence induction, as well as sets of genes specific to the initiating quiescence signal [24,25]. Indeed, synchronization procedures have been shown to induce stress responses specific to the synchronization procedure used [26,27,28]. Characterization of quiescent cells from unperturbed populations has been hindered by the lack of a molecular marker to identify living quiescent cells.

The recent development of a sensor for CDK2 activity enables the identification of live cells that are in quiescence [11]. This sensor consists of an mVenus-tagged section of DNA Helicase B (DHB-mVenus) containing CDK2 phosphorylation sites close to a nuclear localization sequence (NLS) and a nuclear export sequence (NES) (S1A Fig). Phosphorylation of the sensor by CDK2 masks the basic residues of the NLS and unmasks the NES, causing translocation of the sensor to the cytoplasm in a manner that is correlated with CDK2 activity. The cytoplasmic:nuclear ratio of this sensor thus serves as a readout for CDK2 activity. Cells early in the cell cycle show nuclear localization of the sensor and low CDK2 activity, whereas cells toward the end of the cell cycle show cytoplasmic localization of the sensor and high CDK2 activity. S phase begins when the cytoplasmic:nuclear ratio of the sensor is approximately 1. Addition of a CDK2 inhibitor at any time during the cell cycle causes an immediate drop in CDK2 activity, visualized by rapid nuclear translocation of the sensor [11].

When single-cell traces of CDK2 activity from asynchronously cycling cells are aligned to the time of mitosis, a bifurcation in CDK2 activity becomes apparent, which corresponds to the proliferation-quiescence cell fate decision [11]. One subset of cells completes mitosis with residual CDK2 activity (cytoplasmic:nuclear ratio of the sensor ≥ 0.5), which then steadily increases over the course of the cell cycle (CDK2^inc^ cells). Another subset of cells completes mitosis with low or no CDK2 activity and enters quiescence (CDK2^low^ cells; cytoplasmic:nuclear ratio of the sensor < 0.5). This quiescence is transient in nature. Indeed, CDK2^low^ cells experience a second cell fate decision in which they can continue to remain quiescent or emerge from quiescence and re-enter the cell cycle. Cells that emerge from quiescence can be identified by a renewed increase in CDK2 activity (CDK2^emerge^ cells). Thus, upon completion of mitosis, cells can become proliferating CDK2^inc^ cells or quiescent CDK2^low^ cells. CDK2^low^ cells can remain CDK2^low^ for variable amounts of time or re-enter the cell cycle by becoming CDK2^emerge^ cells.

Entry into the CDK2^low^ state occurs in all cell lines examined thus far, even under optimal culture conditions (full-growth media at subconfluent densities). While it is known that the bifurcation in CDK2 activity is regulated by p21 [11], our understanding of why cells enter the CDK2^low^ state is incomplete. We recently showed that 50% of the transits through the CDK2^low^ state can be explained by replication errors carried over from the previous (mother) cell cycle [29]. The trigger for entry into the CDK2^low^ state in the other 50% of CDK2^low^ cells remains unknown, but it is possible that these cells are also experiencing an unidentified stress. Because cells enter the CDK2^low^ state without any exogenous trigger, we refer to CDK2^low^ cells that exist under optimal culture conditions as “spontaneously” quiescent, to contrast with other well-established types of quiescence in which cells are “forced” into quiescence (e.g., serum starvation or contact inhibition).

Despite substantial knowledge about the mechanism of cell-cycle transitions, we do not have a clear picture of overall cell-cycle dynamics detailing the rise and fall of protein levels and appearance and disappearance of protein post-translational modifications. In large part, this is because biochemical approaches in synchronized cells typically monitor only a few protein species at low time resolution. Proteomic surveys of the cell cycle have provided a more global view of cell-cycle events in mammalian cells but also suffer from low temporal resolution [26,30]. Furthermore, any method that relies on cell synchronization to enrich for cells at a specific cell-cycle stage is likely to exert stress on cells, which pollutes actual cell-cycle regulation with regulatory mechanisms operative as cells emerge from an arrested state. In addition, bulk analysis approaches blur heterogeneity in cell-cycle behavior, potentially resulting in incorrect interpretations of biological data. In contrast, time-lapse microscopy can offer single-cell measurements at millisecond temporal resolution in asynchronous cells but is limited by the difficulty of designing live-cell fluorescent readouts of multiple cell-cycle regulators and by the challenges of automated image processing and cell tracking. Most recently, immunofluorescence (IF) staining of fixed-cell snapshots has been used to infer cell-cycle kinetics of a handful of proteins [31,32,33], but without distinguishing proliferating from quiescent cells. Given that spontaneously quiescent cells appear in varying proportions in all cycling populations examined thus far, failure to distinguish proliferating cells from spontaneously quiescent cells leads to increased apparent cell-to-cell variability and decreased accuracy in quantifying protein behavior.

Here we combine the best of live-cell microscopy and antibody-based measurement to map key molecular events during cell-cycle progression versus spontaneous cell-cycle exit. By categorizing cells by their CDK2 activity trajectory (CDK2^inc^, CDK2^low^, CDK2^emerge^) and computationally aligning their IF signal as a function of time-since-anaphase, we reduce cell-to-cell variability in protein measurements and eliminate potential artifacts from synchronization procedures. In this way, we identify several unexpected differences in protein levels and modification states between cells that are progressing through the cell cycle and have increasing CDK2 activity (CDK2^inc^ cells) and cells that are quiescent (CDK2^low^). One noteworthy example is Cyclin D, which is well known (and confirmed here in MCF10A cells) to be expressed at low levels in cells forced into quiescence by serum starvation or contact inhibition, but which we show is more abundant in spontaneously quiescent CDK2^low^ cells compared with proliferating CDK2^inc^ cells. We also identified 4 proteins with concentrations that steadily increase or decrease the longer the cells are in spontaneous quiescence. This result suggests that there exists a continuum of quiescence depths. Together, our single-cell data provide a chronology of key events during the active cell cycle and reveal key molecular differences between forced quiescence, spontaneous quiescence, and proliferation.

## Results

### Single-cell methods for characterizing cell-cycle dynamics in unperturbed cells

We used 2 complementary single-cell methods to chronicle the dynamics of key cell-cycle regulators. The first method uses 4-color IF snapshot images to categorize individual cells as G1, S, G2, M, or G0/quiescent. This approach has the advantage of being readily applicable to any cell line without the need to insert fluorescent sensors or perform time-lapse microscopy but does not explicitly carry time-dependent information. By co-staining cells with Hoechst (to measure DNA content) and EdU (a marker for DNA synthesis) [34], we could subdivide the cell cycle into 5 categories (Fig 1B–1D and S1B Fig): cells with 2N DNA content and no EdU incorporation were classified as G0 or G1; cells with near 2N DNA content and intermediate EdU signal were classified as early S phase; cells with high EdU signal were classified as S phase; cells with near 4N DNA content and intermediate EdU signal were classified as late S phase; and cells with 4N DNA content and no EdU incorporation were classified as G2 or M (Fig 1B).

To further distinguish cells in G0 from cells in G1, we co-stained cells with an antibody against phospho-Rb at either Serine 780 or Serine 807/811. These sites are phosphorylated by CDK2 and thus can serve as a fixed-cell readout of CDK2 activity. The phospho-Rb signal is bimodally distributed, representing hypo- and hyper-phosphorylated Rb (Fig 1C). Newly born cells with hypo-phosphorylated Rb were previously shown to be in the CDK2^low^ state, whereas newly born cells with hyper-phosphorylated Rb are in the CDK2^inc^ state [11]. Therefore, EdU-negative cells with 2N DNA content and hypo-phosphorylated Rb are classified here as G0/quiescent, and EdU-negative cells with 2N DNA content and hyper-phosphorylated Rb are classified here as G1 (Fig 1C). To distinguish cells in G2 from cells in M, we used an antibody against phospho-Histone H3 (pHH3), a well-established marker for mitosis. EdU-negative cells with 4N DNA content that were pHH3-negative were classified as G2, and cells that were pHH3-positive were classified as mitotic (Fig 1D). We used 3 fluorescent channels to stain cells with Hoechst, EdU, and either phospho-Rb or pHH3 (S1C Fig), and used the fourth channel to measure 1 of 14 proteins of interest in MCF10A human mammary epithelial cells. We also validated our results in Hs68 human foreskin fibroblasts. We avoided use of cancer cell lines, which often have mutations in the core cell-cycle regulatory network.

The second method involves time-lapse microscopy over 24 hours of MCF10A cells expressing Histone 2B (H2B) fused to mTurquoise and the CDK2 sensor fused to mVenus. Immediately after the last frame was taken, cells were fixed with para-formaldehyde, processed for IF, and reimaged. Custom MATLAB-based cell-tracking scripts were used to extract single-cell traces of CDK2 activity, with a custom “jitter correction” to re-register the images before and after IF (see Materials and methods). In this way, we can match each cell’s IF staining to its history. The H2B signal is used to automatically identify the frame of anaphase for each cell, which enables automated alignment of all CDK2 activity traces (and consequently each cell’s IF signal) to each cell’s final anaphase of the movie.

The resulting plot demonstrates the bifurcation in CDK2 activity that is evident as cells complete mitosis and assume either a CDK2^inc^, CDK2^low^, or CDK2^emerge^ state (Fig 1E) [11]. Another subset of cells has no mitoses during the course of the 24-hour movie, of which a further subset has low CDK2 activity for the entire 24-hour imaging period. Although these cells are not cycling, they are also not senescent (S2A–S2C Fig) and thus appear to be in a prolonged quiescence (Fig 1F). Indeed, we confirmed that these cells can emerge from this prolonged quiescence (S2D Fig). In our unperturbed MCF10A cells, 95.6% ± 5.4% of the total population divided at least once during the 24-hour imaging. Of the total population,79.0% ± 6.2% entered the CDK2^inc^ state after mitosis, 8.8% ± 2.6% remained CDK2^low^ after mitosis, and 7.8% ± 1.7% entered the CDK2^low^ state after mitosis but built up their CDK2 activity before the end of the imaging period (CDK2^emerge^) (Fig 1E). Among the 4.4% that did not divide during the course of the movie, 52.3% ± 17.7% stayed in a prolonged quiescence (representing 2.3% ± 0.8% of the total population, Fig 1F) and 47.7% ± 17.7% (or 2.1% ± 0.8% of the total population) were observed to build up CDK2 activity before the end of the imaging period (S1 Movie and S2D Fig). For CDK2^emerge^ cells, automated identification of the time point when cells begin building up CDK2 activity after being in a CDK2^low^ state allows automated alignment of CDK2^emerge^ traces to this event (Fig 1G), which we have previously argued represents the R-point [11,35]. Alignment of the CDK2 activity traces in these various ways allows for the staging of IF-based protein levels or modification states as a function of time-since-anaphase, or time-since-R-point.

Cells treated with EdU for 15 minutes at the end of a 24-hour time-lapse sequence illustrate the power of this approach—CDK2^inc^ cells display the classic “rainbow” pattern of EdU as a function of time-since-anaphase, allowing us to identify and label G1, S, and G2 phases of the cell cycle in our time-lapse + IF experiments (Fig 1H, blue). CDK2^low^ cells (Fig 1H, red) and prolonged quiescent cells (Fig 1H, purple) display no EdU signal. A moving average through the CDK2^inc^ and CDK2^low^ subpopulations further illustrates the effect (Fig 1I). CDK2^emerge^ cells aligned to the time at which CDK2 activity begins to increase show a pattern similar to the CDK2^inc^ cells but with less clarity due to the difficulty of automating the identification of the first frame of CDK2 activity rise (relative to the easy automatic identification of the first frame of anaphase; Fig 1J). This plot shows that CDK2^emerge^ cells begin S phase at a similar time after the initial CDK2 activity buildup as CDK2^inc^ cells.

**Fig 2:**
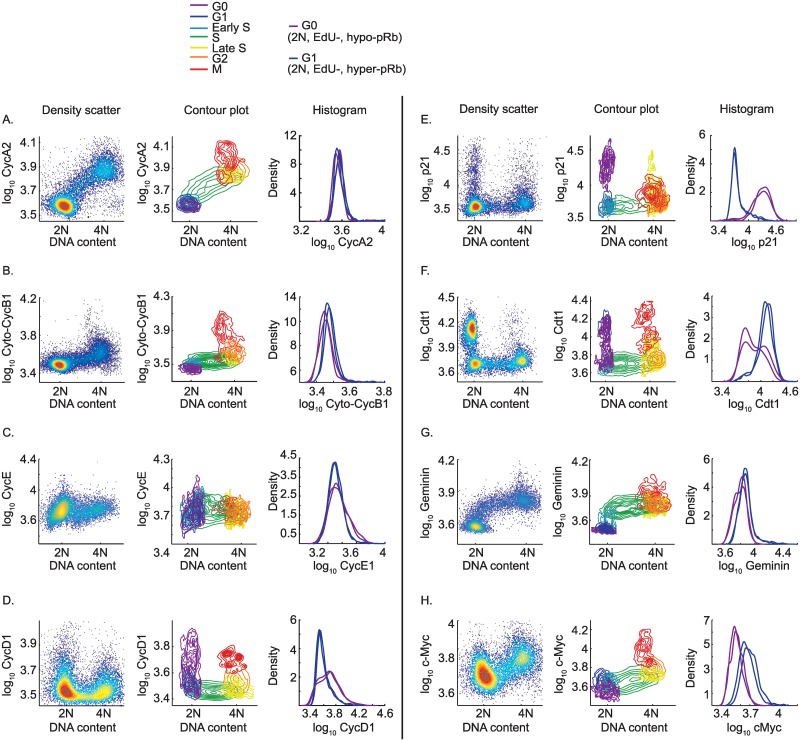
Protein levels for asynchronous MCF10A cells in G0, G1, S, G2, and M phases of the cell cycle. (A–H) Column 1: Density scatter of the indicated protein versus DNA content; data are pooled from 9 IF images from 1 representative well. Column 2: Contour plot of the indicated protein versus DNA content; contours are color-coded by cell-cycle phase according to the legend. Data are pooled from 9 IF images from 1 representative well. Column 3: Histogram (probability density) of the indicated protein for G0 cells (purple, defined as 2N DNA content, EdU-negative, and hypo-phosphorylated Rb) versus G1 cells (blue, defined as 2N DNA content, EdU-negative, and hyper-phosphorylated Rb). Two biological replicates are shown; each replicate represents 9 pooled IF images. Note that the y-axes for Column 1 and Column 2, and the x-axis for Column 3, are in log base 10; units are arbitrary fluorescence units. All signals are nuclear except where indicated. Also note that because of cell rounding during mitosis, IF signal intensities are artificially high in mitotic cells. Abbreviation: Cyto-CycB1, Cytoplasmic Cyclin B1 signal; IF, immunofluorescence; Rb, retinoblastoma protein.

**Fig 3:**
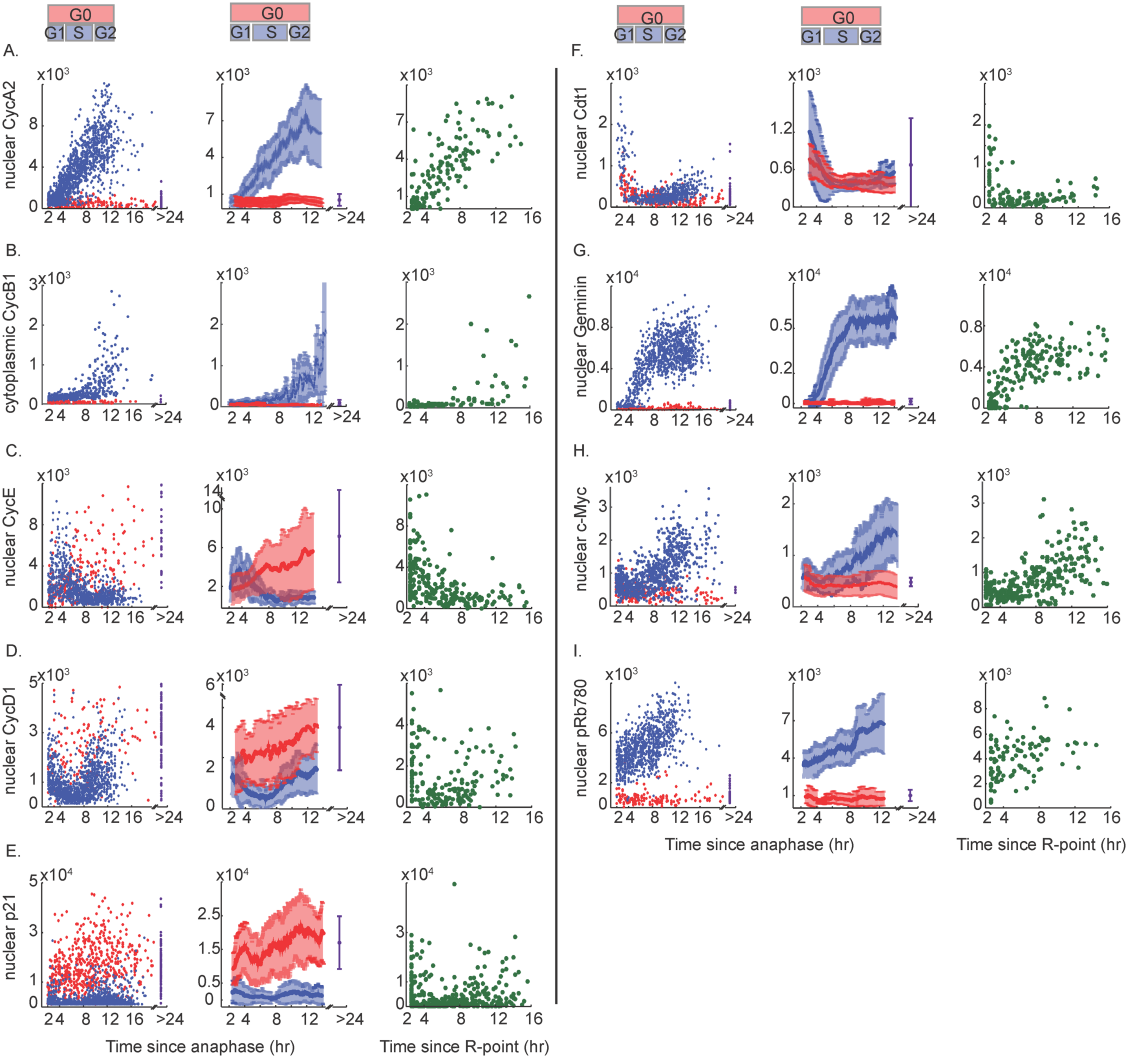
Protein dynamics for proliferating and spontaneously quiescent MCF10A cells. (A–I) Column 1: Time-lapse imaging of CDK2 activity in asynchronous cells was followed by fixation and IF staining for the indicated protein. Protein signals were then reconstructed as a function of time-since-anaphase for CDK2^inc^ cells (blue dots) and CDK2^low^ cells (red dots), as in Fig 1H. The protein signal in prolonged quiescent cells is plotted at the 24-hour mark (purple dots). The approximate time spent in G1, S, or G2 is marked above the plots, based on EdU incorporation data from Fig 1H. Column 2: Moving average through the blue, red, or purple points from Column 1, as in Fig 1I. Error bars represent standard deviation. Column 3: Protein signal as a function of time since CDK2 activity buildup (R-point), as in Fig 1J. The y-axis units are arbitrary fluorescence units. Total number of cells plotted: (A) 1,949; (B) 433; (C) 1,549; (D) 1,729; (E) 2,596; (F) 1,043; (G) 1,885; (H) 2,117; (I) 1,300. The data for each antibody are pooled from 8 replicate wells (1 image per well). Abbreviations: CDK2, Cyclin-Dependent Kinase 2; IF, immunofluorescence; R-point, Restriction Point.

**Fig 4:**
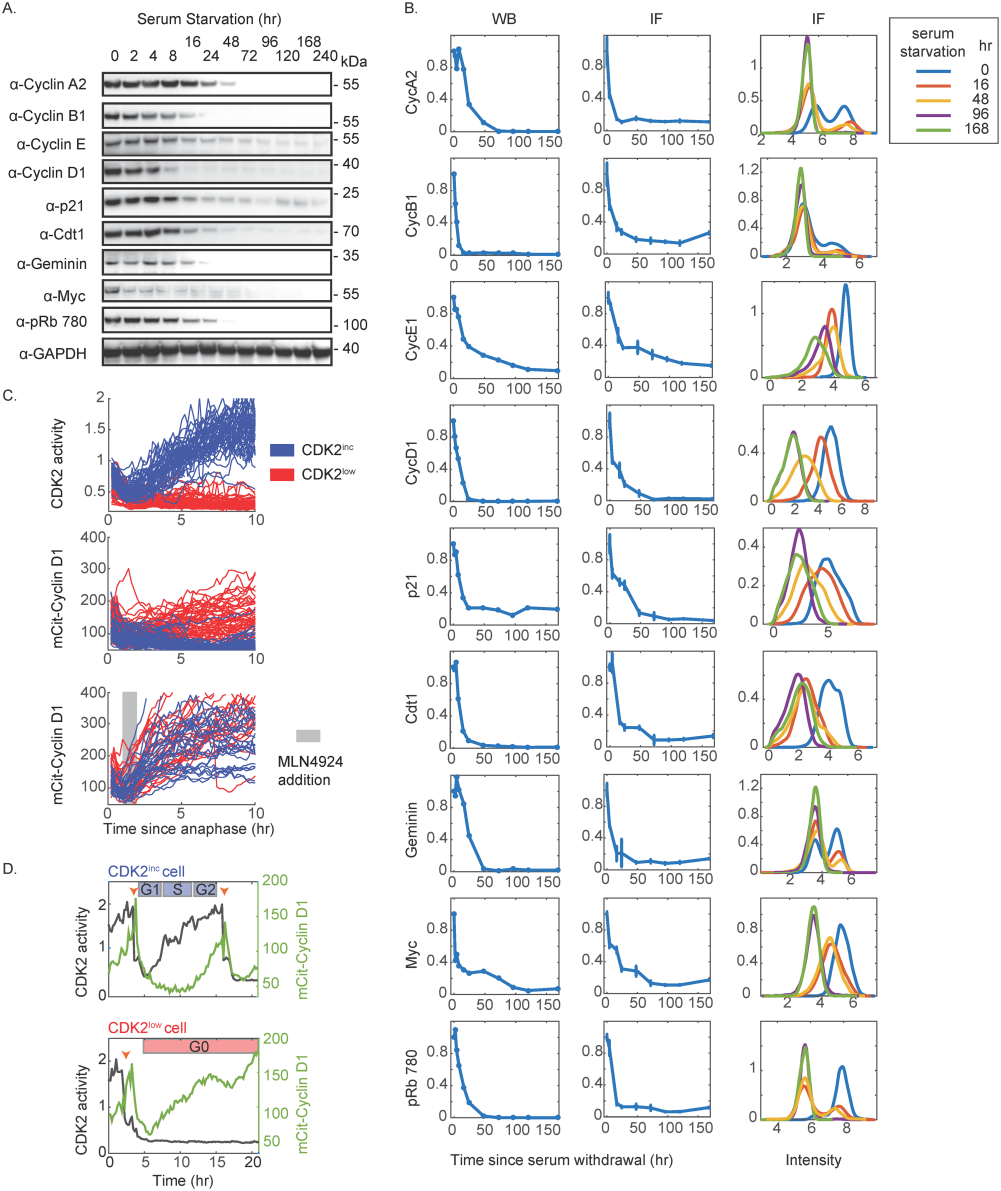
Validation of antibody staining in serum-starved cells and validation of Cyclin D1 results by time-lapse imaging of mCitrine-Cyclin D1. (A–B) Cells were serum starved for the indicated time and analyzed by western blotting (A) or IF (B). (B) Column 1: Quantification of western blots in (A). Column 2: Average IF signal of the indicated antibody signal across 3 replicate wells upon serum starvation for the amounts of time indicated in (A). Error bars represent standard deviation from 3 replicate wells. Column 3: Probability density of the indicated antibody signal at 0, 16, 48, 96, and 168 hours after serum withdrawal. (C) Time-lapse imaging of asynchronous mCitrine-Cyclin D1 knock-in MCF10A cells expressing H2B-mTurquoise and mCherry-tagged CDK2 sensor. Single cell traces of CDK2^inc^ and CDK2^low^ cells are colored blue and red, respectively, and computationally synchronized to anaphase onset. Upper panel: traces of CDK2 activity in control treatment. Middle panel: corresponding traces of mCitrine-Cyclin D1 level in control treatment. Lower panel: traces of mCitrine-Cyclin D1 level in cells that received 1.4 μM MLN4924 at 1-2 hours after anaphase onset. (D) Examples of single-cell traces of CDK2 activity and mCitrine-Cyclin D1 in 1 CDK2^inc^ cell (top) and 1 CDK2^low^ cell (bottom). In CDK2^inc^ cells, mCitrine-Cyclin D1 levels are moderate in G1, fall in S phase, and increase again in G2, whereas mCitrine-Cyclin D1 levels begin to increase steadily shortly after mitosis in CDK2^low^ cells. Orange arrows mark mitoses. Abbreviations: CDK2, Cyclin-Dependent Kinase 2; IF, immunofluorescence; R-point, Restriction Point.

**Fig 5:**
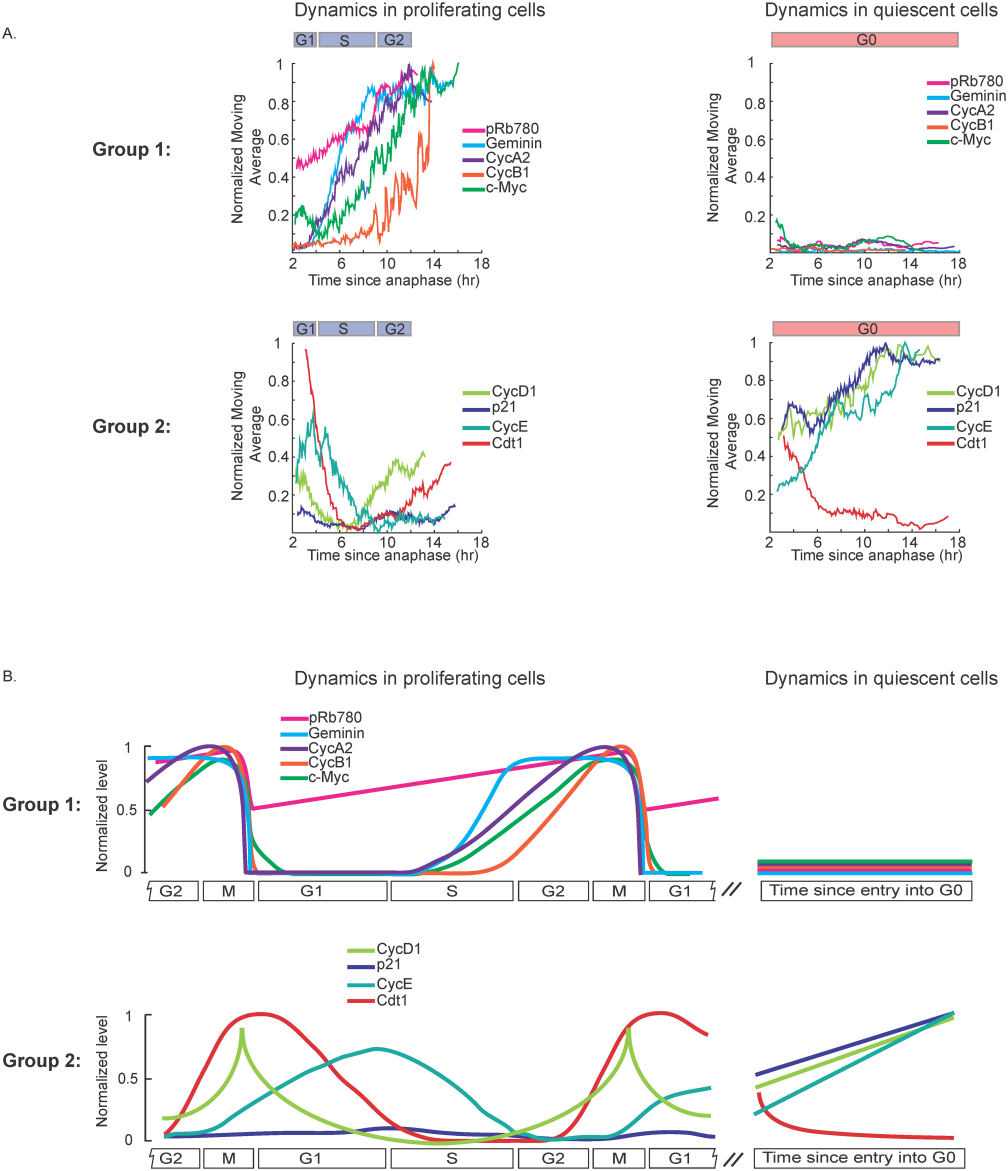
Data synthesis to generate maps of protein dynamics during cell-cycle progression and cell-cycle exit. (A) Moving average traces from Fig 3 Column 2 were normalized such that the minimum signal experienced across CDK2^inc^ and CDK2^low^ data for a given protein was set to 0, and the maximum signal experienced across CDK2^inc^ and CDK2^low^ data for this protein was set to 1. The approximate time spent in G1, S, or G2 phases is marked above the plots, based on EdU incorporation data from Fig 1H. Group 1 (top row): protein signals that are “off” in quiescent cells. Group 2 (bottom row): protein signals that change dynamically over time in quiescent cells. (B) Schematized representation of the data from (A).

These 2 methods were used to chronicle the dynamics of 14 proteins during cell-cycle progression and spontaneous quiescence. The proteins were chosen because of the availability of selective antibodies, their role as core cell-cycle regulators (Cyclin A2, Cyclin B1, Cyclin E, Cyclin D1, p21, p27, Cdt1, Geminin, total Rb, and phospho-Rb), or as important signaling inputs to the cell cycle (cMyc, Fra1, phospho-cJun, and p53). Nine proteins were highly dynamic over the course of the cell cycle (Cyclin A, Cyclin B, Cyclin E, Cyclin D, p21, Cdt1, Geminin, cMyc, and phospho-Rb; Figs 2–5 and S3 Fig), whereas others tested were relatively invariant over the cell cycle in the cell types examined here (p27, total Rb, p53, Fra1, and phospho-cJun; S4 and S5 Figs).

### Characterization of the dynamics of a core cell-cycle network

Using multi-color IF in MCF10A cells, we began by inferring the dynamics of various cell-cycle proteins using (1) a density scatter plot of signal intensity versus DNA content (Fig 2, Column 1); (2) a contour plot of signal intensity versus DNA content, in which cells are grouped into 7 cell-cycle phases, as described in Fig 1B–1D and S1B Fig (Fig 2, Column 2); and (3) a histogram of signal intensity in G0/quiescent cells (2N, EdU-negative, hypo-phosphorylated Rb) versus G1 cells (2N, EdU-negative, hyper-phosphorylated Rb), as defined in Fig 1C (Fig 2, Column 3). We also repeated these experiments in a second cell type, non-immortalized Hs68 human foreskin fibroblasts (S3 and S4 Figs).

Cyclin A2 and Cyclin B1, two of the best-understood cell-cycle proteins, behaved in textbook fashion and serve as a proof-of-principle. In cycling cells, Cyclin A2 rose linearly during S and G2, consistent with previous reports (Fig 2A, scatter and contour plots; and Fig 3A) [31,32,33,36,37]. Cyclin B1 levels did not begin to rise until late S but then rose supra-linearly, as previously reported (Fig 2B, scatter and contour plots; and Fig 3B) [16,31,32]. Cyclins A2 and B1 were both degraded in mitosis [38,39], and both were undetectable in G0 and G1 cells (Fig 2A and 2B, histograms; and Fig 3A and 3B).

When Cyclin E levels were plotted against DNA content, we detected a subtle “N”-shaped pattern in which Cyclin E rose in G1 and fell in S phase, as expected (Fig 2C, scatter plot; [31,36,40,41]). The rise in G1, and fall in early S phase, of Cyclin E is also detected in the time-lapse + IF data for CDK2^inc^ cells (Fig 3C). In contrast with Cyclins A2 and B1, which remained “off” in CDK2^low^ cells, Cyclin E levels rose steadily in CDK2^low^ cells (Fig 3C). This is surprising because Cyclin E is overexpressed in several cancers and Cyclin E/CDK2 activity is a major driver of cell-cycle progression [42]. Therefore, we expected Cyclin E levels to be lower in spontaneously quiescent cells compared with proliferating cells. We note, however, that these high levels of Cyclin E in G0/quiescent cells are not accompanied by high CDK2 activity and thus are not able to stimulate cell-cycle progression; by definition, we identify these quiescent cells because of their lack of CDK2 activity (CDK2^low^). This lack of CDK2 activity despite high levels of Cyclin E is likely due to the accompanying high levels of p21 in these cells (see below). Thus, a likely explanation for the high levels of Cyclin E in G0/quiescent cells may be that Cyclin E in these cells has not been subjected to S phase–mediated degradation, which depends on CDK2 activity [40,41]. We also observed that the Cyclin E antibody utilized here, the widely used clone HE12, detects a strong nonspecific signal in MCF10A cells, in addition to detecting Cyclin E (S6A and S6B Fig). Thus the difference in Cyclin E signal between CDK2^low^ and CDK2^inc^ cells may be partly obscured by the nonspecific signal.

The patterns displayed by Cyclin D1 were also unexpected. MCF10A cells express Cyclin D1, D2, and D3, with Cyclin D1 at the highest level of the three [43]. Thus Cyclin D1 is the prevalent D-type cyclin in our cells, and the antibody used in this study is selective for Cyclin D1 (S6A and S6B Fig). When Cyclin D1 levels were plotted against DNA content, we detected a “U”-shaped pattern in which Cyclin D1 is high in cells with 2N DNA content, low in S phase, and elevated again in cells with 4N DNA content (Fig 2D, scatter and contour plots). This pattern has been reported previously [44,45] but is not widely appreciated. Upon closer inspection, the EdU-negative cells with 2N DNA content reveal highly heterogeneous expression of Cyclin D1—cells with hypo-phosphorylated Rb (G0 cells) have much higher levels of Cyclin D1 than cells with hyper-phosphorylated Rb (G1 cells) (Fig 2D, histogram). Like Cyclin E, this is surprising because Cyclin D is considered a driver of the cell cycle and is overexpressed in several cancers [46]; therefore, its levels are expected to be higher in proliferating cells than in quiescent cells. When we examined our time-lapse + IF data, we observed the same phenomenon—cells born into the quiescent CDK2^low^ state had high Cyclin D1 levels, whereas CDK2^inc^ cells that were actively progressing through the cell cycle again displayed a “U”-shaped pattern, with Cyclin D1 levels being moderate in G1, low in S phase, and moderate again in G2 (Fig 3D). In addition, prolonged quiescent cells also have high Cyclin D1 levels (Fig 3D, purple).

By way of explanation, we considered the possibility that Cyclin D1 levels appear higher in G0 cells simply because Cyclin D1 in these cells has not been subjected to S phase-mediated degradation [47,48]. However, the moving average of Cyclin D1 levels indicated that CDK2^low^ cells have higher levels of Cyclin D1 than CDK2^inc^ cells, even in cells 2 hours after birth, before S phase-mediated degradation could play a role. We also note that CDK2^low^ cells have more Cyclin D1 than CDK2^inc^ cells ever have, at least on average. However, high levels of Cyclin D do not necessarily correspond to high CDK4/6 activity [49,50], and there is as yet no single-cell assay to measure CDK4/6 activity in these cells. An alternative explanation for the high Cyclin D1 levels in CDK2^low^ cells is that Cyclin D1 protein levels are stabilized by high levels of p21 in these cells [51,52,53].

Indeed, p21 displays the same “U”-shaped pattern as Cyclin D1 does when plotted against DNA content (Fig 2E, scatter and contour plots). As with Cyclin D1, cells with hypo-phosphorylated Rb (G0/quiescent cells) have high levels of p21, whereas EdU-negative, 2N DNA content with hyper-phosphorylated Rb (G1/proliferating cells) have very low levels of p21 (Fig 2E, histogram). Moreover, time-lapse + IF data revealed that p21 levels are high in newly born G0/CDK2^low^ cells and very low in newly born G1/CDK2^inc^ cells, as reported previously (Fig 3E) [11]. CDK2^emerge^ cells show initially high levels of p21 that then decay around the time that CDK2 activity turns back on (Fig 3E, green), consistent with the notion that a decay in p21 enables a rise in CDK2 activity.

CDK2^inc^ cells maintain very low levels of p21 throughout all of G1 and S phase (Fig 2E, contour plot; and Fig 3E, blue). While these data are consistent with our previous studies [11,29], these results differ from the common notion that p21 levels are generally high in G1 cells [54]. A likely explanation for this discrepancy is that many previous studies used various treatments (e.g., nocodazole or serum starvation) for cell synchronization, which exert stress on cells and can increase p21 levels [55,56]. Furthermore, immunoblotting does not allow fine-grained analysis of p21 heterogeneity or temporal behavior. More recent single-cell experiments tracking exogenous YFP-p21 in U2OS osteosarcoma cells detected newly born cells with and without YFP-p21 [57]. However, without a live-cell marker to distinguish G0 from G1, it is not possible to know if the newly born cells with elevated p21 are actually passing through a G0/CDK2^low^ state rather than going straight to G1. Similarly, the cells born without detectable p21 could represent a G1/CDK2^inc^ subpopulation.

The dynamics of Cdt1 are expected to have some similarities to p21 because both proteins are substrates of the E3 ubiquitin ligase CRL4^Cdt2^ [13], a feature reflected in our IF data (Fig 2F, scatter and contour plots). However, in direct contrast to p21, Cdt1 levels are high in G1 cells and lower in G0/quiescent cells (Fig 2F, histogram). Time-lapse + IF data show a similar trend, revealing that any residual Cdt1 present in CDK2^low^ cells is quickly degraded to the basal level seen in S phase cells (Fig 3F). The levels of Geminin, an inhibitor of Cdt1, are out of phase with Cdt1, as expected [13,58,59]. Geminin levels are undetectable in quiescent CDK2^low^ cells and begin to rise in early S phase, consistent with Geminin’s role as a substrate of the APC/C (Figs 2G and 3G) [35,58]. However, unlike Cyclin A2, which rises steadily and linearly, Geminin levels plateau by mid-to-late S phase, a feature seen in both IF and time-lapse + IF data, suggesting an additional layer of transcriptional regulation.

We also examined the cell-cycle dynamics of c-Myc, a key protein that links MAPK signaling to cell-cycle entry [60]. More recently, c-Myc has been shown to act as a “transcription amplifier” as opposed to a classic transcription factor [61,62]. Here we show that c-Myc is strongly cell-cycle regulated. Immunofluorescence reveals that c-Myc levels are higher in G1 cells than in G0/quiescent cells (Fig 2H, histogram), consistent with a pro-proliferation role for c-Myc. CDK2^low^ cells maintain low c-Myc levels as long as they remain in the CDK2^low^ state but then up-regulate c-Myc upon emerging from the CDK2^low^ state (Fig 3H). c-Myc levels rise steadily in S and G2 phases (Fig 2H, scatter and contour plots; and Fig 3H).

Phospho-Rb is bimodally distributed among EdU-negative cells with 2N DNA content (Fig 1C) [11]. The switch from hypo- to hyper-phosphorylated Rb marks passage through the R-point [9,63,64], and while this event is often cited as occurring in mid- to late G1 [9,63], we have shown previously that MCF10A cells are born into a state of either hypo- or hyper-phosphorylated Rb immediately upon completion of mitosis [11]. Here we extend this result by confirming that the same is true using an antibody against Rb phosphorylation at another site, Serine 780 (Fig 3I)—cells born into the quiescent CDK2^low^ state have hypo-phosphorylated Rb, whereas cells born into the cell cycle-committed CDK2^inc^ state have hyper-phosphorylated Rb. This phospho-Rb-S780 signal continues to rise as CDK2^inc^ cells progress through the cell cycle. Examination of the CDK2^emerge^ cells provides additional information by revealing that cells present with hyper-phosphorylated Rb as soon as the rise in CDK2 activity can be detected, indicating that hyper-phosphorylation of Rb occurs prior to or concurrently with activation of CDK2 (Fig 3I, green).

### Comparison of spontaneous quiescence with quiescence induced by serum starvation or contact inhibition

Given the surprising behavior of several proteins in spontaneous quiescence (e.g., rising Cyclin D1, Cyclin E, and p21 levels), we compared our results in spontaneously quiescent cells with quiescence induced by well-established methods, namely serum starvation (Fig 4A and 4B) and contact inhibition (S6C Fig). By both quantitative western blotting and IF, we were able to reproduce the canonical protein dynamics upon serum starvation or contact inhibition in which the levels of Cyclin D1, Cyclin E, p21, and all other proteins examined, fell as a function of time in quiescence. We also validated the selectivity of the antibodies used for IF via siRNA knockdown (S6A and S6B Fig) and provide sample images for each IF stain (S7 Fig).

### Validation of Cyclin D1 behavior

We next sought to further validate the unexpected dynamics of Cyclin D1 using an antibody-independent method. We used CRISPR-mediated genome editing of MCF10A cells to tag Cyclin D1 at its endogenous locus with mCitrine, a yellow fluorescent protein, and subsequently transduced the cells with H2B-mTurquoise and mCherry-tagged CDK2 sensor. Western blotting and PCR revealed that both alleles of Cyclin D1 were tagged with mCitrine (S8A and S8B Fig) and IF revealed a linear correlation at the single-cell level between the mCitrine-Cyclin D1 signal and an antibody stain against Cyclin D1 (S8C Fig). In agreement with our time-lapse + IF results for Cyclin D1, single-cell tracking of the mCitrine-Cyclin D1 cell line showed that CDK2^low^ cells have elevated Cyclin D1 levels compared with CDK2^inc^ cells (Fig 4C red traces, and Fig 4D bottom panel), and that the levels of Cyclin D1 for CDK2^inc^ cells are moderate in G1, low in S phase, and moderate again in G2 (Fig 4C blue traces, and Fig 4D top panel). These results explain why Cyclin D1 expression was recently reported to be a poor predictor of the time spent between mitosis and S phase [45].

Given that c-Myc levels are low and Rb is hypo-phosphorylated (and thus that E2F transcription is inhibited) in the spontaneously quiescent CDK2^low^ cells, what factors could be driving the high levels of Cyclin D1? Since these 2 major cell-cycle transcription factors are likely off in CDK2^low^ cells, we hypothesized that the high Cyclin D1 levels in these cells could be due to a lack of degradation. Indeed, Cyclin D1 levels are strongly regulated not only by transcription but also by protein degradation via cullin-RING ligases (SCF with various F-box proteins) [65]. Because the majority of cullin-RING ligases require covalent modification by NEDD8 for holoenzyme ubiquitin ligase activity, their activity can be inhibited by blocking their neddylation with the small molecule MLN4924 [66]. We therefore filmed mCitrine-Cyclin D1 cells before and after an acute treatment with 1.4 μM of MLN4924 and selected for analysis only those cells that received drug 1–2 hours after mitosis (during G0/G1). Consistent with our hypothesis, inhibition of cullin-RING ligases caused an increase in Cyclin D1 in CDK2^inc^ cells to a level that was comparable with that in CDK2^low^ cells. Thus, lack of Cyclin D1 degradation in CDK2^low^ cells is a major contributor to the high levels of Cyclin D1 seen in these cells.

Cyclin D1 is well known for its short half-life. These results suggest that the stability of Cyclin D1 varies with cell-cycle phase—the half-life of Cyclin D1 is short in CDK2^inc^ cells but much longer in CDK2^low^ cells. Together, these validation experiments lend confidence in our overall approach and in the unexpected findings in this work.

### Synthesis of data to generate maps of cell-cycle dynamics

To compare the relative protein dynamics in proliferating versus quiescence cells, we normalized and overlaid the moving average data from Fig 3 for CDK2^inc^ and CDK2^low^ cells (Fig 5A; note that normalizing the signals masks differences in dynamic range among proteins). Proteins were grouped into 2 plots according to their behavior in quiescent CDK2^low^ cells—Group 1 contains signals that are “off” in quiescent cells (Fig 5A, top), and Group 2 contains signals that change dynamically over time in quiescent cells (Fig 5A, bottom). The identification of 4 proteins in Group 2 that either steadily increase (Cyclin D1, Cyclin E, p21) or steadily decrease (Cdt1) the longer a cell has been quiescent suggests that quiescence is not just a single static state but rather that certain aspects of a cell’s proteome evolve as a function of time spent in quiescence (at least over the 24-hour period that we measured).

We then schematized these results to create diagrams that depict the chronology and dynamics of cell-cycle events (Fig 5B). The 5 proteins in Group 1 all increase their levels as cells progress through the proliferation cycle, albeit with different dynamics. Cyclin A2 starts to accumulate in S phase and continues to increase until M phase. Geminin begins to accumulate at the same time but plateaus in G2. Cyclin B1 and c-Myc remain low until late S phase. Rb phosphorylation on Serine 780 steadily increases throughout the whole proliferative cycle. All of these proteins reset at mitosis and maintain low levels in quiescent cells. The dynamics of Group 2 proteins are more variable. Cyclin D1 and Cdt1 turn on in G2 after being low or off in S phase. Cyclin D1 increases further if cells go into G0, and degrades when CDK2^inc^ cells re-enter the cell cycle. Cdt1 decreases slowly when cells enter the CDK2^low^ state and decreases rapidly when CDK2^inc^ cells enter S phase. Cyclin E starts to increase at the completion of mitosis and continues to increase throughout G0 and G1; Cyclin E levels drop because of degradation at the G1/S transition but remain elevated in G0/quiescent cells. p21 levels are low in proliferating cells but increase steadily once cells enter quiescence. Such diagrams provide a quantitative resource for understanding the dynamics of cell-cycle proteins relative to one another.

## Discussion

Using single-cell time-lapse microscopy and IF, combined with automated image processing and cell tracking, we have characterized the dynamics of key cell-cycle proteins in unperturbed proliferating and spontaneously quiescent cells and compared these with cells forced into quiescence by serum starvation or contact inhibition. Our measurements provide a rich resource for those focused on the cell cycle, or on any biological process that is impacted by the cell cycle, by providing a map of standard cell-cycle behavior in non-tumorigenic cells. Unlike most characterizations of cell-cycle behavior, which use chemical synchronization such as nocodazole or double thymidine block, our data come from asynchronous, unperturbed single cells. We are therefore able to chart, at high time resolution, both mean population behavior as well as cell-to-cell variability in protein levels and modification states. All cultured populations of somatic human cells that we have examined thus far actually contain mixtures of proliferating and spontaneously quiescent cells. This generates extensive cell-to-cell variability, which would obscure even single-cell IF data aligned by time-since-anaphase, if one were unable to distinguish the proliferating, quiescent, and emerging populations using the CDK2 sensor.

When we classified proteins based on their behavior in quiescent CDK2^low^ cells, we identified a set of 4 proteins (Cyclin D1, Cyclin E, p21, and Cdt1), whose concentrations increase or decrease the longer cells are in quiescence. This suggests that quiescence is not a homogenous “off” state, but rather that the quiescent cell state changes continually, at least over our 24-hour observation period. These data support the existence of a continuum of quiescence depths.

It is well documented that the levels of Cyclins D and E are dramatically reduced in cells forced into quiescence via serum starvation [42,67]. Here we compared the dynamics of multiple key cell-cycle proteins, including Cyclin D1 and Cyclin E, in forced versus spontaneous quiescence. In contrast to the declining levels of Cyclin D1 and Cyclin E in serum-starved or contact-inhibited cells, the levels of Cyclin D1 and Cyclin E rise while cells are in the quiescent CDK2^low^ state. We confirmed this result using endogenously tagged mCitrine-Cyclin D1 and further showed that the high levels of Cyclin D1 in CDK2^low^ cells arise because of reduced cullin-RING ligase-mediated protein degradation in the CDK2^low^ state. Similarly, high levels of Cyclin E in CDK2^low^ cells likely arise because this Cyclin E has not been subjected to S phase–mediated degradation, which depends on CDK2 activity [40,41].

Examination of cells emerging from a transient quiescence (CDK2^emerge^ cells) reveals that CDK2^emerge^ cells recapitulate the protein dynamics of cells that immediately enter the CDK2^inc^ state after mitosis. Put another way, the protein dynamics of CDK2^inc^ cells that are born committed to the cell cycle with elevated CDK2 activity are similar to the protein dynamics of CDK2^emerge^ cells that commit to the cell cycle at variable times after dividing. This result argues that the beginning of the active cell cycle is marked by the increase in CDK2 activity and that any time cells spend prior to the activation of CDK2 represents a period of cell-cycle exit that we have referred to as G0/quiescence.

Which signaling events are causes of, and which are simply consequences of, entry into quiescence? The full answer will require extensive analysis using acute perturbations of the proteins in question, but based on the data presented here, we can already speculate that the behavior of proteins with levels that are similar in newly born CDK2^inc^ and CDK2^low^ cells is likely to simply be a consequence of entry into quiescence (e.g., Geminin, Cyclin A2, and Cyclin B1). In contrast, proteins with levels that are already distinct in newly born CDK2^inc^ and CDK2^low^ cells have already been shown to be causative (e.g., p21 [11]) or have the potential to be causative in the proliferation-quiescence decision (e.g., Cyclin D1 and phospho-Rb).

In summary, the experimental and computational approaches employed here enable the creation of chronological maps of protein dynamics during cell-cycle progression and cell-cycle exit in asynchronous single cells, revealing several differences compared with previous results generated from synchronized cells. These maps will be informative for mathematical modeling of the cell cycle and can also serve as a benchmark for comparing the cell cycle of non-transformed cells with the cell cycle of various cancer cells. Our work also highlights the fact that there are multiple molecularly distinct states of quiescence, depending on the initiating trigger. Together, our data provide new information for answering fundamental questions about normal cellular control over proliferation and add new molecular knowledge to the poorly documented state(s) of G0/quiescence.

## Materials and methods

### Cell culture and reagents

MCF10A human mammary epithelial cells were maintained in DMEM/F12 (ThermoFisher) supplemented with 5% horse serum (Invitrogen), 20 ng/ml epidermal growth factor (EGF, Sigma-Aldrich), 0.5 mg/ml hydrocortisone (Sigma-Aldrich, St. Louis, MO), 100 ng/ml cholera toxin (Sigma-Aldrich), 10 μg/ml insulin (Invitrogen), and penicillin/streptomycin. For serum starvation media, the horse serum, EGF, and insulin were removed, and 0.3% BSA was added. For live-cell time-lapse imaging, phenol-red free DMEM/F12 was used. Hs68 primary human foreskin fibroblasts were cultured in DMEM with 10% FBS and penicillin/streptomycin. Both cell lines were purchased from ATCC. Hs68 cells can be propagated for 42 passages according to ATCC and are not immortalized; cells were received at passage 12 and were used within 13 passages of receipt. MCF10A cells expressing the CDK2 sensor (DHB-mVenus) and tagged histone H2B (H2B-mTurquoise) are as described [11].

Integration of the mCitrine-encoding gene into the CCND1 locus was carried out using CRISPR technology [68]. A CRISPR-Cas9 ribonucleoprotein (RNP) complex was generated using the CRISPR-Cas9 System from IDT. The RNP contains crRNA (GGAGCUGGUGUUCCAUGGCUGUUUUAGAGCUAUGCU) annealed to tracrRNA and Cas9 nuclease. The RNP was electroporated into MCF10A cells using the Neon system from Life Technologies following the manufacturer’s protocol with 2 pulses, 30 ms at 1150 V. Single cells were sorted by flow cytometry into 96-well plates and grown into clones. Western blot and IF against Cyclin D1 protein, as well as PCR of the *CCND1* gene, were carried out as validation. Data from clone 2A7 is shown in this work (S8 Fig). For functional validation, cells were treated with Mek inhibitor (PD0325901, S1036 from Selleckchem) at 100 nM for 32 hours, or treated for 32 hours followed by a Mek inhibitor washout for 6 hours. To confirm a similar response to inhibition of degradation for both mCitrine-Cyclin D1 and endogenous Cyclin D1, the mCitrine-Cyclin D1 line and parental wild type MCF10A line were treated with MLN4924 (Active Biochem, A-1139) at 1.4 μM or Bortezomib (Cayman Chemical,10008822) at 1 μM for 2 hours (S8A Fig).

### siRNA

siRNA oligos were synthesized by Dharmacon: CCNA2 (MU-003205-02-002), CCNE1 (MU-003213-02-0002), CCNE2 (MU-003214-02-0002), CDKN1A (MU-003471-00-0002), CCND1 (MU-003210-05-0002), RB1 (MU-003296-03-0002) or IDT: CCNB1 (hs.Ri.CCNB1.13.1), GMNN (hs.Ri.GMNN.13.1), CDT1 (hs.Ri.CDT1.13.2), MYC (hs.Ri.MYC.13.2), and Negative Control DsiRNA (51-01-14-04). The oligos were electroporated into MCF10A cells following manufacturer’s instruction (Neon system, Life Technologies). Cells were fixed for IF or lysed for western blotting 20 hours (for short-live proteins: Cyclin A2, Cyclin B1, Cyclin E, Cyclin D1, c-Myc, p21, Geminin, and Cdt1) or 48 hours (for longer-live proteins: Rb) after the electroporation.

### Immunofluorescence

Antibodies used in this study are p21 Waf1/Cip1 (CST #2947) at 1:250, phospho-Rb (Ser807/811) (CST #8516) at 1:250, phospho-Rb 780 (BD Biosciences #668385) at 1:250, p21 (BD Biosciences #556430) at 1:250, total Rb (a gift from Julien Sage) at 1:200, p53 (DO-1) (Santa Cruz sc-126) at 1:100 and p53 (Ab-1) (Calbiochem OP03) at 1:100, Fra-1 (Santa Cruz #28310) at 1:200, Cyclin E clone HE12 (Zymed #32–1600) at 1:400, Cyclin D1 clone SP4 (Thermo Scientific RM-9140-S0) at 1:250, Cyclin A2 (Santa Cruz #751) at 1:500, phospho-Histone H3 (Ser10) (CST #9706 and #9701) at 1:200, p27 (BD Bioscience #610241) at 1:100, Geminin (CST #5165) at 1:250, Cyclin B1 (CST #4138) at 1:100, c-Myc (CST #5605) at 1:250, CDT1 (CST #8064) at 1:200, phospho-c-Jun (Ser73) (CST #3270) at 1:800, and Alexa Fluor-488, -546, -647 secondary antibodies (ThermoFisher) at 1:500.

For Cyclin E IF, cells were fixed in −20°C methanol for 5 minutes and then washed twice with PBS. For all other antibodies, cells were fixed with 4% paraformaldehyde and then washed twice with PBS. Cells were then incubated with a blocking/permeabilization buffer (10% FBS, 1% BSA, 0.1% TX-100 and 0.01% NaN_3_ for antibodies against Cyclin E, p21, Cdt1, Geminin, Fra1, p53, and phospho-c-Jun) for an hour at room temperature, or sequentially permeabilized with 0.2% TX-100 for 15 minutes at 4°C and blocked with 3% BSA for an hour at room temperature (for antibodies against Cyclin A2, Cyclin B1, Cyclin D1, c-Myc, p27, and total Rb). Primary antibody staining was carried out overnight at 4°C in the corresponding blocking buffer and visualized using secondary antibodies conjugated to Alexa Fluor-488, -546, or -647. Where phospho-Rb and phospho-Histone H3 antibodies were used in conjunction with an antibody for a protein of interest in Fig 2, cells were processed using the method appropriate for the protein of interest. Where indicated, cells were incubated in media containing 10 μM EdU for 15 minutes, and then fixed and processed according to manufacturer’s instructions (ThermoFisher #C10340).

Images were acquired on an ImageXpress Micro XLS widefield microscope (Molecular Devices) with a 10X 0.45NA objective and processed using custom scripts in MATLAB.

### Time-lapse microscopy

Cells were plated at least 24 hours prior to imaging in phenol red-free full-growth media in a 96-well plate (Greiner bio-one #655090) such that the density would remain subconfluent until the end of the imaging period. Images were acquired every 12 minutes on an ImageXpress Micro XLS widefield microscope (Molecular Devices) with a 10X 0.45NA objective; CFP exposure = 75 ms; YFP exposure = 200 ms. Cells were imaged in a humidified, 37°C chamber at 5% CO_2_.

### Image processing and cell tracking

Images were processed as described in Cappell et al., 2016 ([35]), with a general description reproduced here: Mean nuclear intensities were measured by averaging the background-subtracted pixel intensities in each nucleus as defined by a nuclear mask. The nuclear mask was established by performing segmentation on H2B-mTurquoise- or Hoechst-stained images as follows. Log-transformed images were convolved with a rotationally symmetric Laplacian of Gaussian filter and objects were defined as contiguous pixels exceeding a threshold filter score. In order to segment cells in contact with their nearest neighbor, a custom segmentation algorithm was implemented to detect and bridge concave inflections in the perimeter of each object (hereafter referred to as the “deflection bridging algorithm”). The deflection bridging algorithm was implemented on every identified object in the first imaging frame and then only adaptively in subsequent frames. This was accomplished by iteratively tracking cells in each frame, detecting probable merge events (as discussed below) and selectively implementing the deflection bridging algorithm on putative merged objects. Local background subtraction was performed on images of sensors or antibodies that were nuclear in subcellular distribution. For local background subtraction, the nuclear mask was expanded by 25 μm and the background for each cell was calculated as the median pixel intensity of local nonmasked pixels. For cytoplasmically localized sensors or antibodies, the nuclear mask was dilated by 50 μm, and the global background was calculated as the mode intensity of all nonmasked pixels. As before, CDK2 activity was calculated as the ratio of cytoplasmic to nuclear mean DHB fluorescence, with the cytoplasmic component calculated as the mean of the top 50th percentile of a ring of pixels outside of the nuclear mask. Tracking of cells between frames was implemented by screening the nearest future neighbor for consistency in total H2B-mTurquoise fluorescence (“conservation of mass”).

Because the stage jittered slightly after fixation and IF in the time-lapse + IF dataset, we implemented the following jitter correction procedure to ensure precise matching of the CDK2 activity trace of each cell to its IF intensity: We first subtracted the image at a specific time from the image in the next frame to get a “difference score” between 2 images. We then repeated the process, with 1 image moving in a 2-dimensional manner, to get multiple “difference scores” when the stage jittered. The position with the lowest score indicated the amount of jittering and the images were aligned accordingly.

“Conservation of mass” was further exploited to detect merges or splits, which allowed recovery of overlapping traces. Mitosis events (called at anaphase) were called when the total H2B fluorescence of the 2 nearest future neighbors of a given cell were both between 45% and 55% of the total H2B fluorescence of the past cell. The R-point was defined as the time CDK2 activity first began to rise. Computationally, this involves calculating slopes of CDK2 activity using windows of 6–10 time points and then maximizing a linear function for time-since-mitosis, CDK2 activity, and CDK2 slope (long times-since-mitosis, low CDK2 activity, and high CDK2 slope).

The tracking code is available for download here: https://github.com/scappell/Cell_tracking.

### Definition of populations

Traces were computationally classified, and manually verified, as CDK2^inc^ (blue), CDK2^low^ (red), or CDK2^emerge^ (green) based on CDK2 activity at 2 hours after mitosis: CDK2^inc^ traces must remain ≥ 0.5 for all frames post-anaphase; CDK2^low^ traces must remain < 0.5 for all frames post-anaphase; CDK2^emerge^ traces initially enter the CDK2^low^ state and then emerge—these traces must remain < 0.5 for at least 3 hours post-anaphase before rising.

## Supporting information

S1 Fig Methods for measuring cell-cycle progression, or lack thereof. (A) The CDK2 sensor consists of an mVenus-tagged peptide containing 4 CDK2 phosphorylation sites (S) close to an NLS and an NES. Phosphorylation of the sensor by CDK2 masks the basic residues of the NLS and unmasks the NES, and causes translocation of the sensor to the cytoplasm in a manner correlated with CDK2 activity. The cytoplasmic:nuclear ratio of this sensor thus serves as a readout for CDK2 activity. See Spencer et al., 2013 [11] for details. (B) Defining cells in different cell-cycle phases using multiple markers. Cutoffs were defined conservatively to select a relatively pure population of the cells of interest; see Fig 1B–1D for the gates (cutoffs) used. (C) Dye and filter cubes used to visualize the IF signals. Abbreviations: CDK2, Cyclin-Dependent Kinase 2; IF, immunofluorescence; NES, nuclear export sequence; NLS, nuclear localization sequence; POI, protein of interest.
(PDF)

S2 Fig Quiescent, CDK2^low^ MCF10A are not senescent and can reengage with the cell cycle. (A) MCF10A were stained for β-galactosidase activity after being exposed to sustained vehicle control (DMSO, 5 days, left), Nutlin-3 (8 μM, 5 days, middle), or Etoposide (12.5 μM, 24-hour treatment followed by drug washout and 4 days of recovery in growth media, right). DMSO-treated cells do not stain positive for β-galactosidase activity compared with cells treated with either Nutlin-3 or Etoposide. Scale bar, 50 μm. (B) The β-galactosidase activity stain was quantified by first examining each channel (red, green, and blue) of the RGB images; cells that stained turquoise for β-galactosidase activity had low values in the red channel. We therefore manually outlined each cell in the images shown using a custom MATLAB GUI and stored the red pixel values for each cell. The red pixel values for each cell were then plotted as histograms. Ten cells’ histograms are shown for each condition; the total number of cells analyzed is indicated in (C). Given the bimodality of the β-galactosidase activity stain in some cells treated with Nutlin-3, we used the saddle point (50 AU, red dashed line) as a threshold for blueness (equivalent to a lack of redness) and counted the number of cells with at least 5% of their red pixels as below this value. (C) Table depicting the number of senescent cells in each image based on the quantification in (B). No DMSO-treated cells had 5% of their pixels below the 50 AU threshold, whereas the Nutlin-3– and Etoposide-treated cells had 41% and 81% below this threshold, respectively. (D) Cells can re-enter the cell cycle after a prolonged period in the CDK2^low^ state. The plot shows CDK2 activity traces from individual unperturbed MCF10A cells that started the movie in the CDK2^low^ state, emerged from the CDK2^low^ state at some point in the movie, and did not have a mitosis during the imaging period. The percentage of the total population with this behavior is indicated; error represents the standard deviation across 96 replicate wells. Abbreviation: CDK2, Cyclin-Dependent Kinase 2.
(PDF)

S3 Fig Protein levels for asynchronous Hs68 cells in G0, G1, S, G2, and M phases of the cell cycle. (A–H) Column 1: Density scatter of the indicated protein versus DNA content; data are pooled from 9 IF images from 1 representative well. Column 2: Contour plot of the indicated protein versus DNA content; contours are color coded by cell-cycle phase according to the legend. Data are pooled from 9 IF images from 1 representative well. Column 3: Histogram (probability density) of the indicated protein for G0 cells (purple, defined as 2N DNA content, EdU-negative, and hypo-phosphorylated Rb) versus G1 cells (blue, defined as 2N DNA content, EdU-negative, and hyper-phosphorylated Rb). Two biological replicates are shown. Abbreviations: IF, immunofluorescence, Rb, retinoblastoma protein.
(PDF)

S4 Fig Levels of proteins that are relatively invariant in asynchronous MCF10A and Hs68 cells. Column 1: Density scatter of the indicated protein versus DNA content. Column 2: Histogram (probability density) of the indicated protein for G0 versus G1 cells (as defined in Fig 1C). Two biological replicates are shown.
(PDF)

S5 Fig Dynamics of proteins that are relatively invariant in proliferating and spontaneously quiescent MCF10A cells. Column 1: Time-lapse imaging of CDK2 activity in asynchronous cells was followed by fixation and IF staining for the indicated protein. Protein signals were then reconstructed as a function of time since anaphase for CDK2^inc^ cells (blue dots) and CDK2^low^ cells (red dots), as in Fig 1H. Nuclear intensity for Cyclin B1 is included as a comparison to the cytoplasmic intensity for Cyclin B1 shown in Fig 3. We include data from 2 widely used antibodies for p53, one which shows no difference between CDK2^inc^ and CDK2^low^ cells and the other which shows p53 to be slightly higher in CDK2^low^ cells. Column 2: Moving average through the blue or red points from Column 1. Error bars represent standard deviation. All data are from MCF10A cells. Number of cells plotted: p27: 714; total Rb: 1,462; p53(Ab-1): 1,357; p53(DO-1): 1,897; Fra1: 1,804; Cyclin B1: 318. The data for each antibody come from 8 replicate wells, pooled together. Abbreviations: CDK2, Cyclin-Dependent Kinase 2; IF, immunofluorescence; Rb, retinoblastoma protein.
(PDF)

S6 Fig Validation of antibodies used in this study. (A) MCF10A cells were transfected with siRNAs against the indicated genes for 20 hours (for CCNA2, CCNB1, CCNE1/2, CDKN1A, CDT1, GMNN, and MYC) or 48 hours (for RB1) and analyzed by western blot. Note only the bottom band on the anti-Cyclin E blot is specific for Cyclin E. (B) Distribution of IF signal intensity after siRNA treatments described in (A). (C) MCF10A cells were contact inhibited for the indicated time and then analyzed by western blot. Abbreviation: IF, immunofluorescence; siRNA, small interfering RNA.
(PDF)

S7 Fig Representative IF images for the antibodies used in this study. Abbreviation: IF, immunofluorescence.
(PDF)

S8 Fig Characterization of the mCitrine-Cyclin D1 knock-in MCF10A cell line. (A) Left: The expected DNA band sizes for the wild-type CCND1 gene and the mCitrine-Cyclin D1 fusion. Right: PCR amplification of the CCND1 gene in parental and mCitrine-CCND1 knock-in cells run in duplicate on 0.8% agarose gel. The mCitrine gene was knocked into both CCND1 alleles resulting in the absence of the wild-type band in the mCitrine-CCND1 knock-in cells. Bands were excised and sequenced as additional verification. (B) The mCitrine-Cyclin D1 knock-in MCF10A cell line responds in the same way as untagged Cyclin D1 in parental MCF10A cells. WT: parental MCF10A cells. CCND1 knock-in: MCF10A cells with mCitrine knocked into the CCND1 locus to produce a mCitrine-Cyclin D1 fusion protein. MLN: 1.4 μM MLN4924 treatment for 2 hours. Btz: 1 μM Bortezomib treatment for 2 hours. Meki: 100 nM PD0325901 treatment for 32 hours. Meki WO: 100 nM PD0325901 treatment for 32 hours followed by drug washout and return to full growth medium for 6 hours. (C) mCitrine intensity linearly correlates with Cyclin D1 antibody staining in the mCitrine-Cyclin D1 knock-in cell line. Left: representative images of mCitrine signal and Cyclin D1 antibody staining in the same cells. Right: Quantification of the images; axes are natural log scale. Abbreviation: WT, wild type.
(PDF)

S9 Fig Data synthesis for proteins that are relatively invariant over the cell cycle. Moving average traces from S5 Fig Column 2 were normalized such that the minimum signal experienced between CDK2^inc^ and CDK2^low^ data for a given protein was set to 0, and the maximum signal experienced between CDK2^inc^ and CDK2^low^ data for this protein was set to 1. Abbreviation: CDK2, Cyclin-Dependent Kinase 2.
(PDF)

S1 Movie CDK2 activity in unperturbed MCF10A cells. MCF10A cells expressing mVenus-tagged CDK2 sensor were imaged in full growth media every 12 minutes for 24 hours. The arrows mark representative cells that enter the CDK2^inc^ (blue), CDK2^low^ (red), or CDK2^emerge^ (green) state after mitosis, or a prolonged quiescent cell (purple) that was quiescent throughout the movie. The CDK2 activity of the 4 cells over time is plotted in the right panel. Abbreviation: CDK2, Cyclin-Dependent Kinase 2.
(AVI)

## Abbreviations

APC/C: anaphase-promoting complex/cyclosome
CDK: Cyclin-Dependent Kinase
IF: immunofluorescence
NES: nuclear export sequence
NLS: nuclear localization sequence
Rb: retinoblastoma protein
R-point: Restriction Point
