# Supplementary material for: A map of protein dynamics during cell-cycle progression and cell-cycle exit
Source: PLoS Biol. 2017 Sep 11;15(9):e2003268. doi: 10.1371/journal.pbio.2003268 (PMC5608403; doi:10.1371/journal.pbio.2003268)
Supplement: S1 Fig — (A) The CDK2 sensor consists of an mVenus-tagged peptide containing 4 CDK2 phosphorylation sites (S) close to an NLS and an NES. Phosphorylation of the sensor by CDK2 masks the basic residues of the NLS and unmasks the NES, and causes translocation of the sensor to the cytoplasm in a manner correlated with CDK2 activity. The cytoplasmic:nuclear ratio of this sensor thus serves as a readout for CDK2 activity. See Spencer et al., 2013 [11] for details. (B) Defining cells in different cell-cycle phases using multiple markers. Cutoffs were defined conservatively to select a relatively pure population of the cells of interest; see Fig 1B–1D for the gates (cutoffs) used. (C) Dye and filter cubes used to visualize the IF signals. Abbreviations: CDK2, Cyclin-Dependent Kinase 2; IF, immunofluorescence; NES, nuclear export sequence; NLS, nuclear localization sequence; POI, protein of interest. (PDF) [file pbio.2003268.s001.pdf]

A.

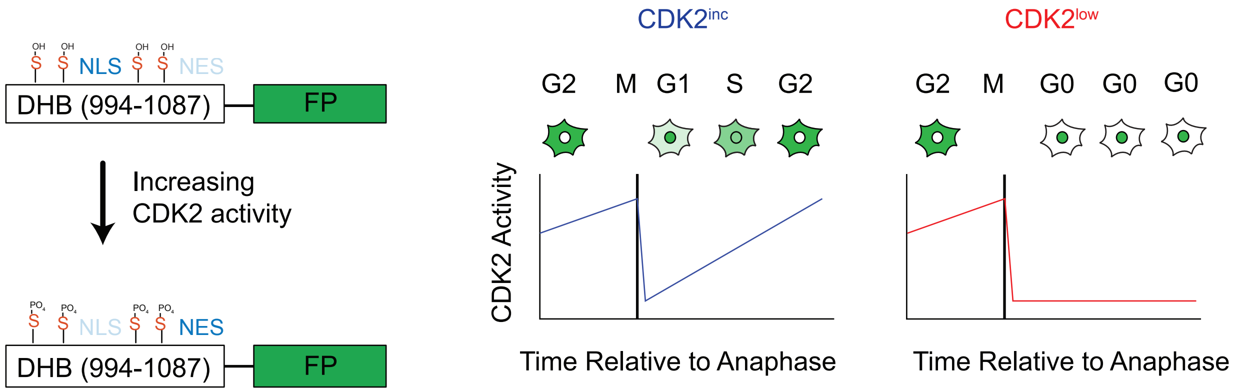

$$\text{CDK2 activity} = \frac{\text{Cytoplasmic signal}}{\text{Nuclear signal}}$$

B.

	DNA	EdU	pRb	pHH3
G0-like	< 2N cutoff	< negative cutoff	< cutoff	
G1	< 2N cutoff	< negative cutoff	> cutoff	
Early s	< 2N cutoff	> negative cutoff<br>< positive cutoff		
S		> positive cutoff		
Late S	> 4N cutoff	> negative cutoff<br>< positive cutoff		
G2	> 4N cutoff	< negative cutoff		< cutoff
M				> cutoff

C.

Signal	Dye	Filter
DNA	Hoechst	DAPI
POI	AF488	FITC
pRb / pHH3	AF546	Cy3
EdU	AF647	Cy5

Supplemental Figure 1.
