# Supplementary material for: A map of protein dynamics during cell-cycle progression and cell-cycle exit
Source: PLoS Biol. 2017 Sep 11;15(9):e2003268. doi: 10.1371/journal.pbio.2003268 (PMC5608403; doi:10.1371/journal.pbio.2003268)
Supplement: S2 Fig — (A) MCF10A were stained for β-galactosidase activity after being exposed to sustained vehicle control (DMSO, 5 days, left), Nutlin-3 (8 μM, 5 days, middle), or Etoposide (12.5 μM, 24-hour treatment followed by drug washout and 4 days of recovery in growth media, right). DMSO-treated cells do not stain positive for β-galactosidase activity compared with cells treated with either Nutlin-3 or Etoposide. Scale bar, 50 μm. (B) The β-galactosidase activity stain was quantified by first examining each channel (red, green, and blue) of the RGB images; cells that stained turquoise for β-galactosidase activity had low values in the red channel. We therefore manually outlined each cell in the images shown using a custom MATLAB GUI and stored the red pixel values for each cell. The red pixel values for each cell were then plotted as histograms. Ten cells’ histograms are shown for each condition; the total number of cells analyzed is indicated in (C). Given the bimodality of the β-galactosidase activity stain in some cells treated with Nutlin-3, we used the saddle point (50 AU, red dashed line) as a threshold for blueness (equivalent to a lack of redness) and counted the number of cells with at least 5% of their red pixels as below this value. (C) Table depicting the number of senescent cells in each image based on the quantification in (B). No DMSO-treated cells had 5% of their pixels below the 50 AU threshold, whereas the Nutlin-3– and Etoposide-treated cells had 41% and 81% below this threshold, respectively. (D) Cells can re-enter the cell cycle after a prolonged period in the CDK2low state. The plot shows CDK2 activity traces from individual unperturbed MCF10A cells that started the movie in the CDK2low state, emerged from the CDK2low state at some point in the movie, and did not have a mitosis during the imaging period. The percentage of the total population with this behavior is indicated; error represents the standard deviation across 96 replicate wells. Abbreviation: CD [file pbio.2003268.s002.pdf]

A.

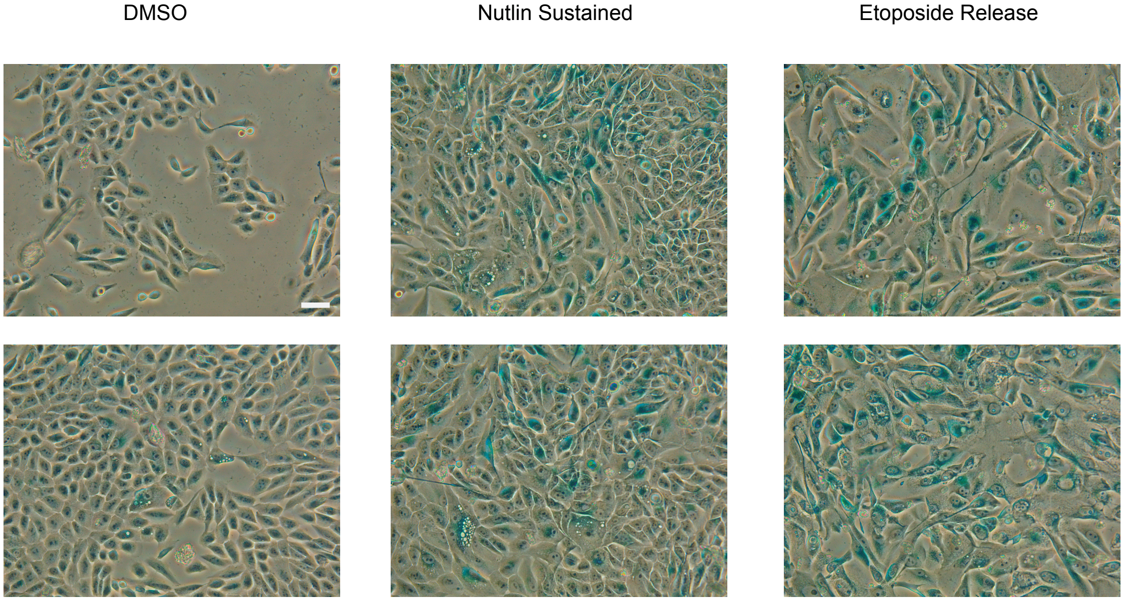

B.

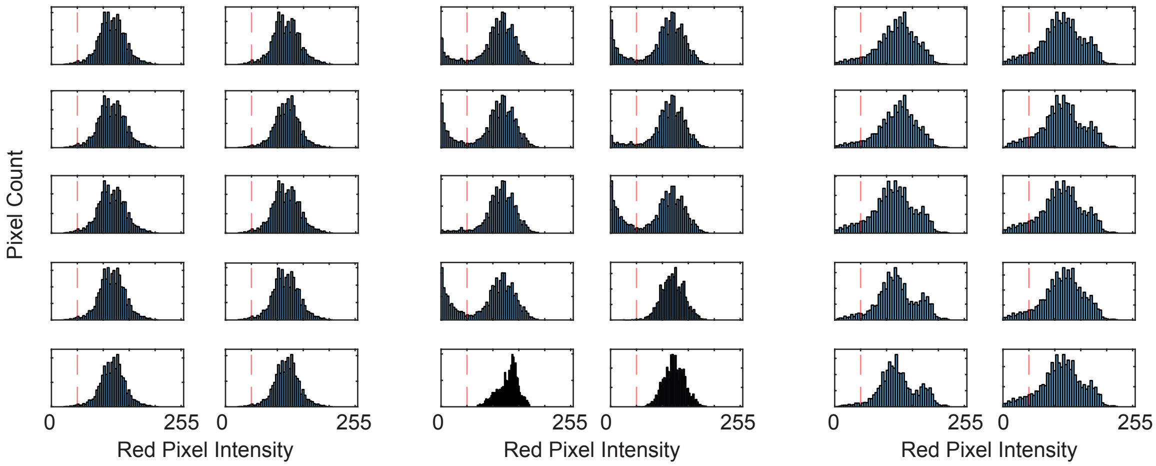

C.

	Total Cells Analyzed	Number Senescent	Percent Senescent
DMSO	195	0	0%
Nutlin Sustained	334	136	41%
Etoposide Release	155	126	81%

D.

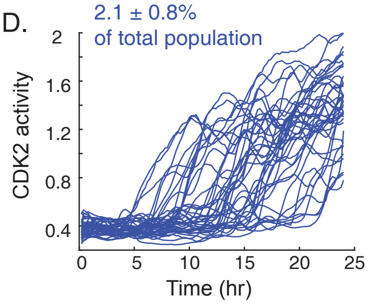

Supplemental Figure 2.
