# Supplementary figures and images for: A map of protein dynamics during cell-cycle progression and cell-cycle exit
Source: PLoS Biol. 2017 Sep 11;15(9):e2003268. doi: 10.1371/journal.pbio.2003268 (PMC5608403; doi:10.1371/journal.pbio.2003268)

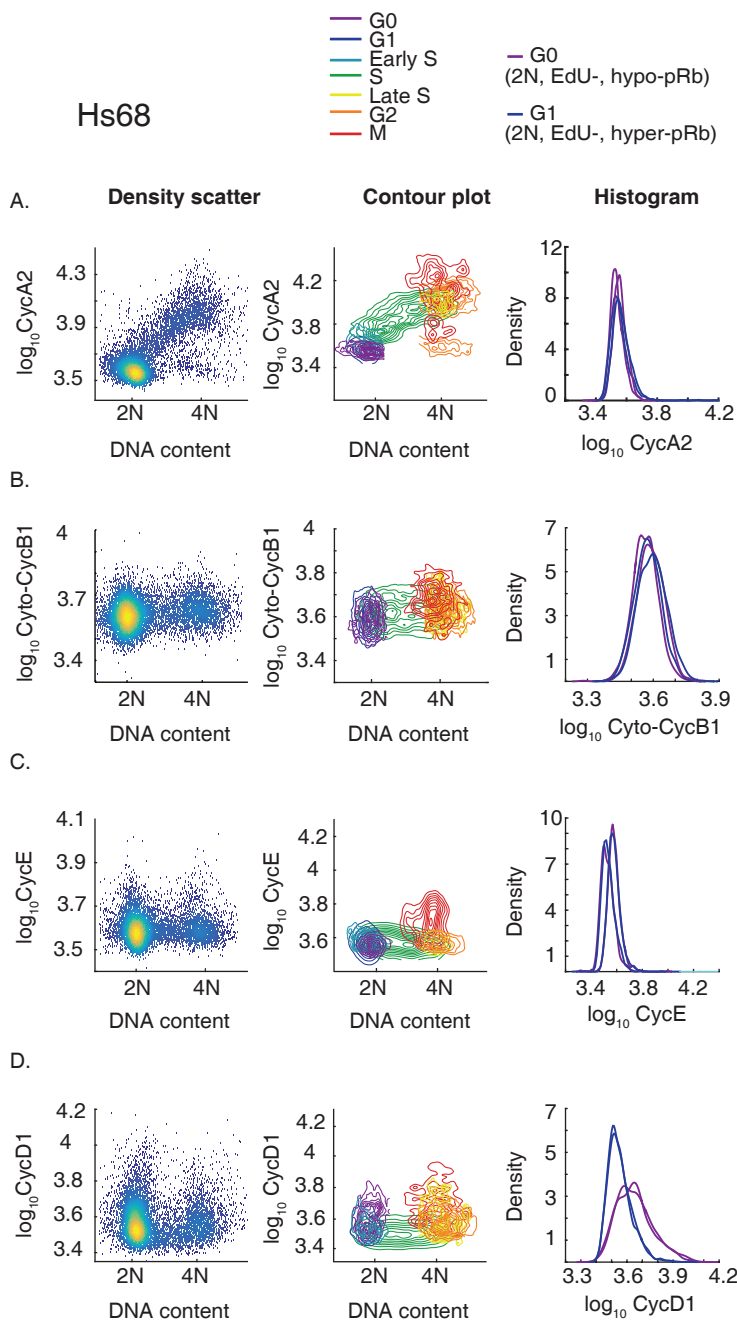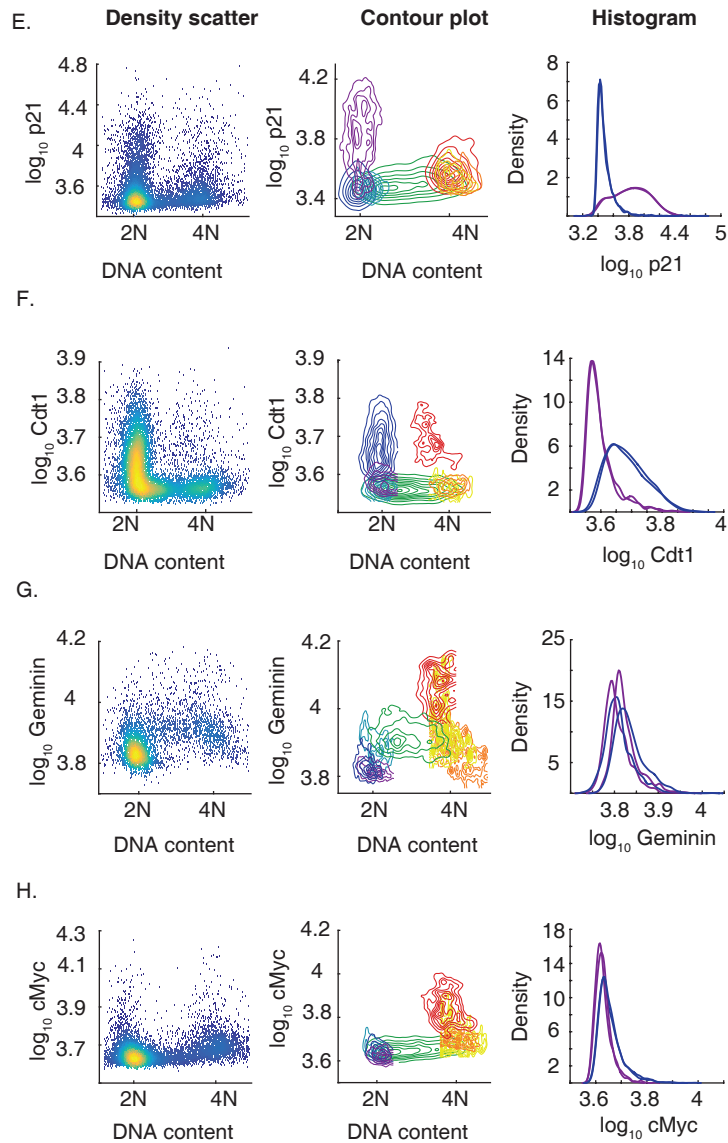

Supplement: S3 Fig — (A–H) Column 1: Density scatter of the indicated protein versus DNA content; data are pooled from 9 IF images from 1 representative well. Column 2: Contour plot of the indicated protein versus DNA content; contours are color coded by cell-cycle phase according to the legend. Data are pooled from 9 IF images from 1 representative well. Column 3: Histogram (probability density) of the indicated protein for G0 cells (purple, defined as 2N DNA content, EdU-negative, and hypo-phosphorylated Rb) versus G1 cells (blue, defined as 2N DNA content, EdU-negative, and hyper-phosphorylated Rb). Two biological replicates are shown. Abbreviations: IF, immunofluorescence, Rb, retinoblastoma protein. (PDF) [file pbio.2003268.s003.pdf]

## MCF10A

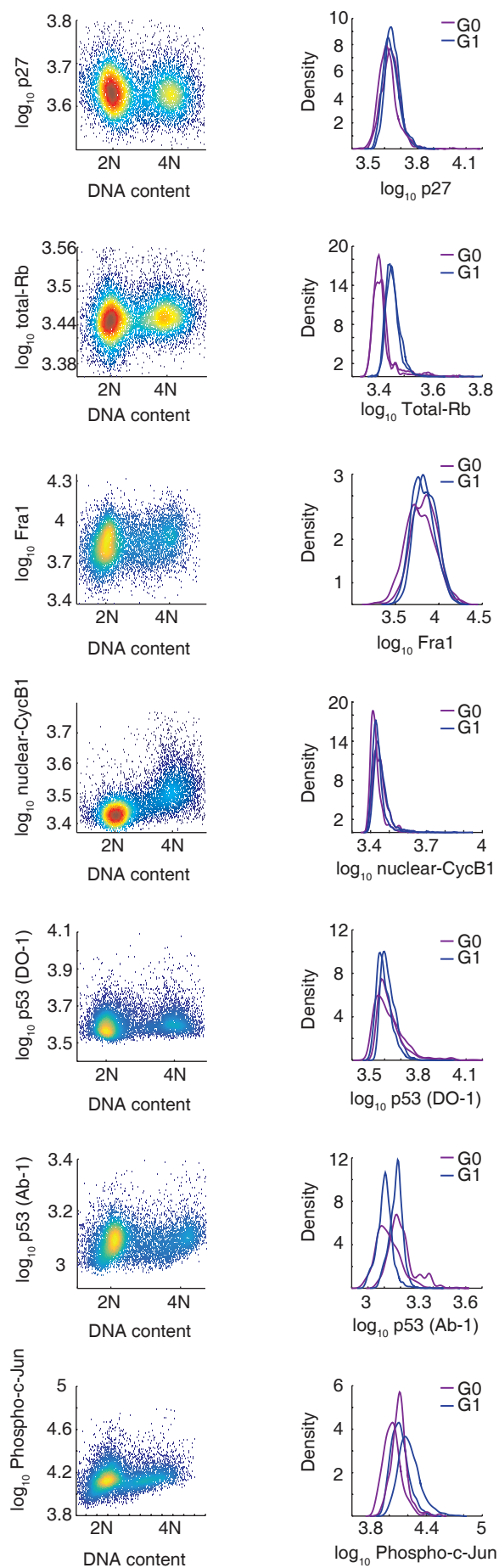

## Hs68

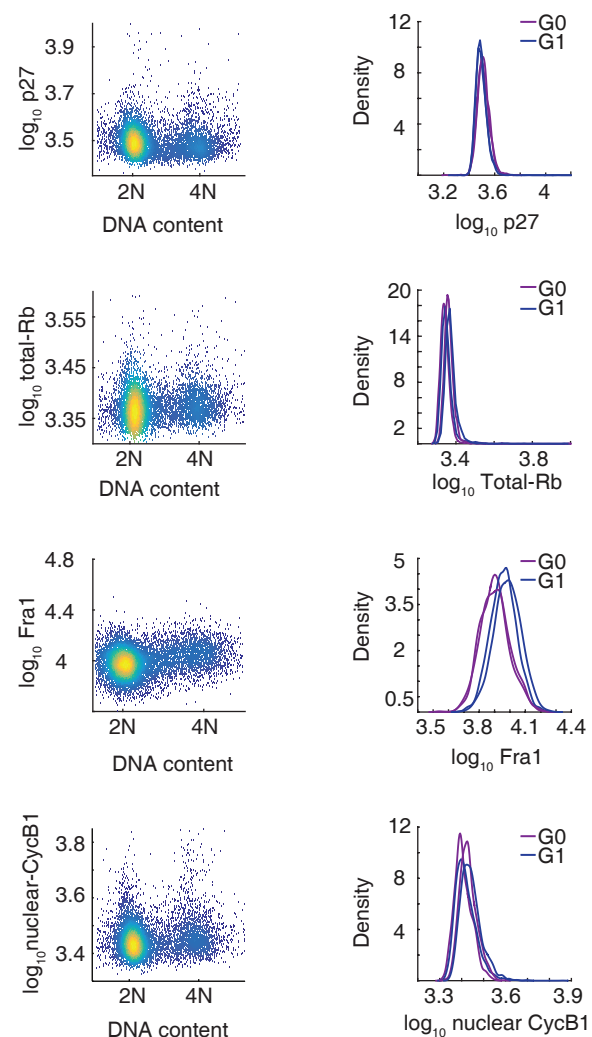

Supplemental Figure 4.

Supplement: S4 Fig — Column 1: Density scatter of the indicated protein versus DNA content. Column 2: Histogram (probability density) of the indicated protein for G0 versus G1 cells (as defined in Fig 1C). Two biological replicates are shown. (PDF) [file pbio.2003268.s004.pdf]

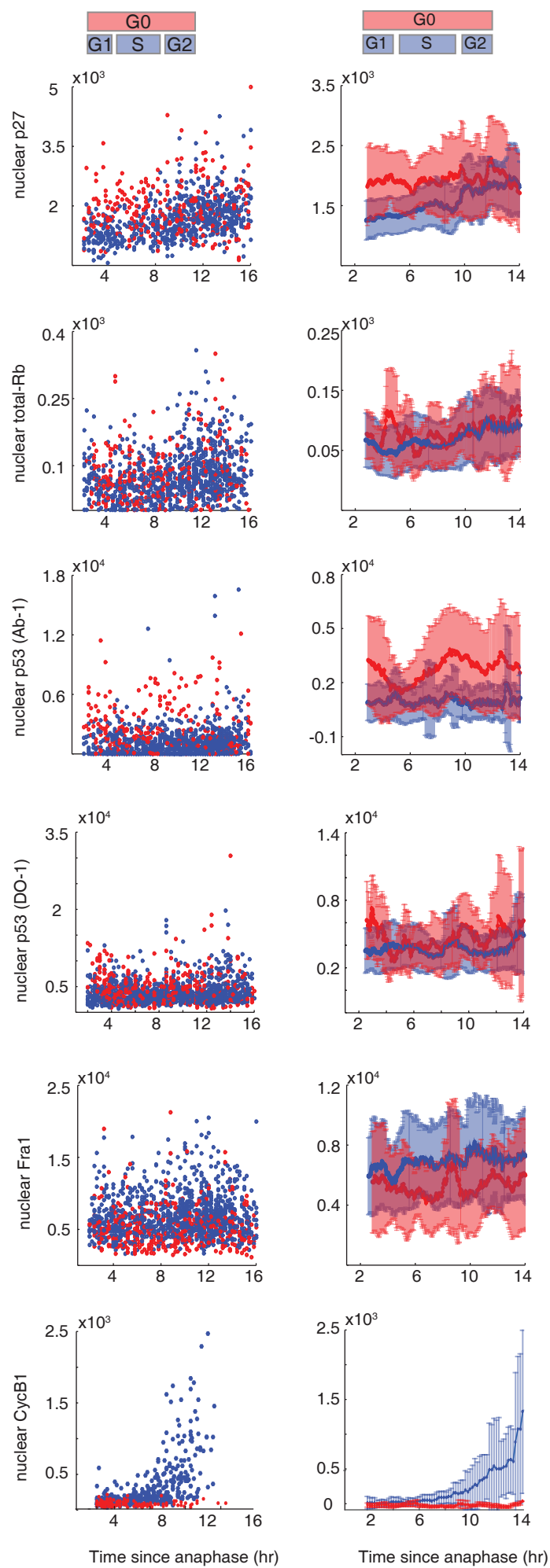

Supplemental Figure 5.

Supplement: S5 Fig — Column 1: Time-lapse imaging of CDK2 activity in asynchronous cells was followed by fixation and IF staining for the indicated protein. Protein signals were then reconstructed as a function of time since anaphase for CDK2inc cells (blue dots) and CDK2low cells (red dots), as in Fig 1H. Nuclear intensity for Cyclin B1 is included as a comparison to the cytoplasmic intensity for Cyclin B1 shown in Fig 3. We include data from 2 widely used antibodies for p53, one which shows no difference between CDK2inc and CDK2low cells and the other which shows p53 to be slightly higher in CDK2low cells. Column 2: Moving average through the blue or red points from Column 1. Error bars represent standard deviation. All data are from MCF10A cells. Number of cells plotted: p27: 714; total Rb: 1,462; p53(Ab-1): 1,357; p53(DO-1): 1,897; Fra1: 1,804; Cyclin B1: 318. The data for each antibody come from 8 replicate wells, pooled together. Abbreviations: CDK2, Cyclin-Dependent Kinase 2; IF, immunofluorescence; Rb, retinoblastoma protein. (PDF) [file pbio.2003268.s005.pdf]

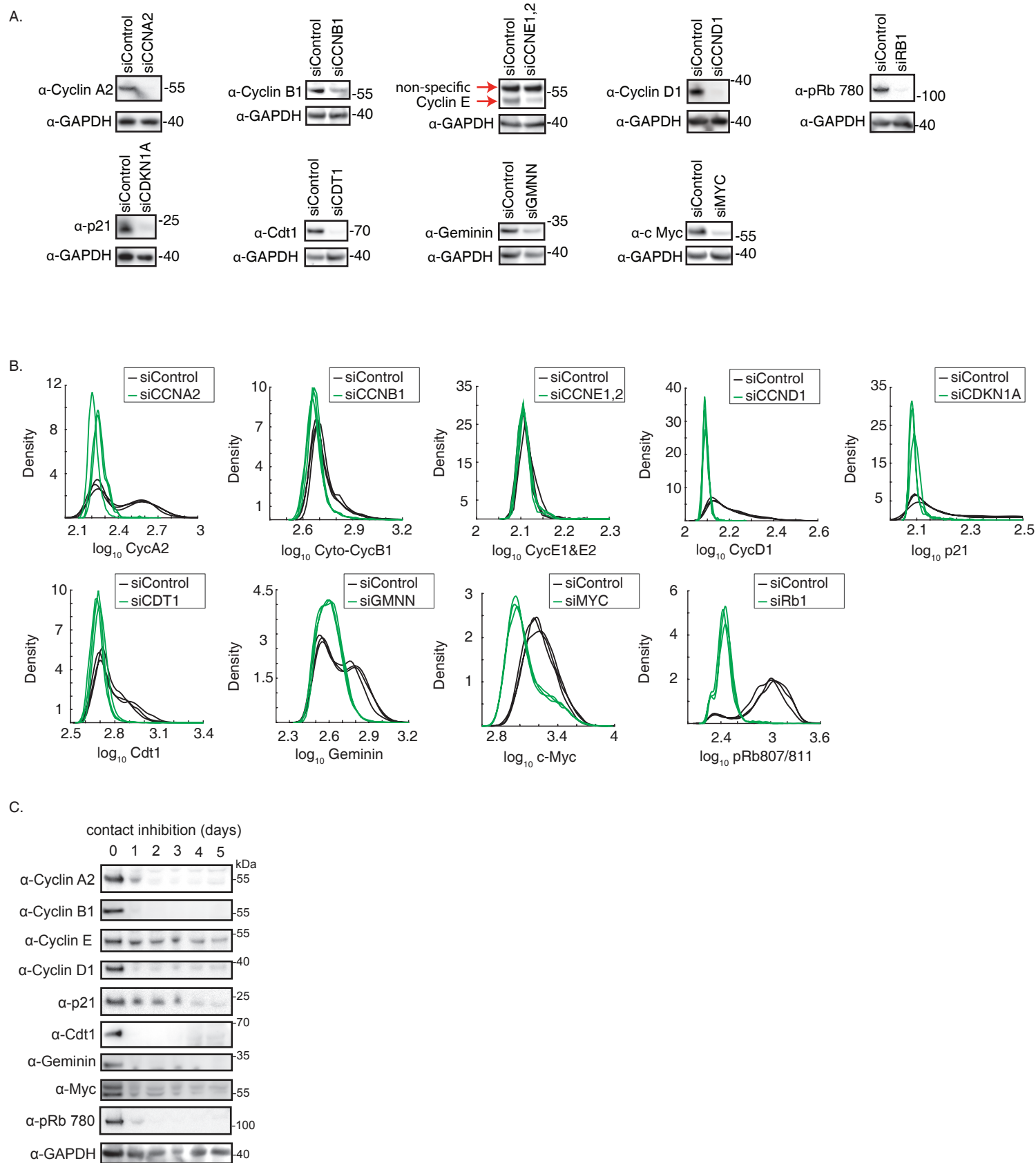

Supplemental Figure 6.

Supplement: S6 Fig — (A) MCF10A cells were transfected with siRNAs against the indicated genes for 20 hours (for CCNA2, CCNB1, CCNE1/2, CDKN1A, CDT1, GMNN, and MYC) or 48 hours (for RB1) and analyzed by western blot. Note only the bottom band on the anti-Cyclin E blot is specific for Cyclin E. (B) Distribution of IF signal intensity after siRNA treatments described in (A). (C) MCF10A cells were contact inhibited for the indicated time and then analyzed by western blot. Abbreviation: IF, immunofluorescence; siRNA, small interfering RNA. (PDF) [file pbio.2003268.s006.pdf]

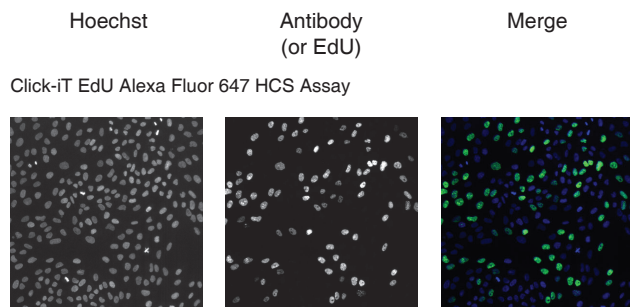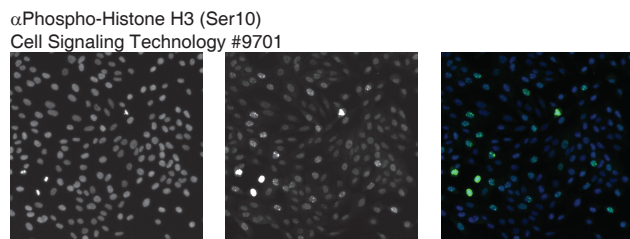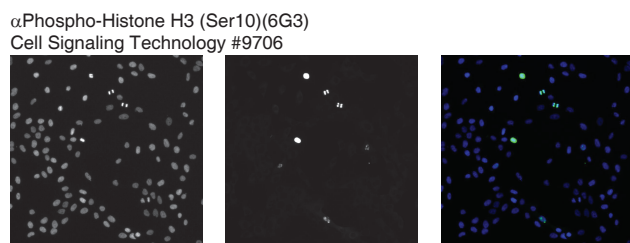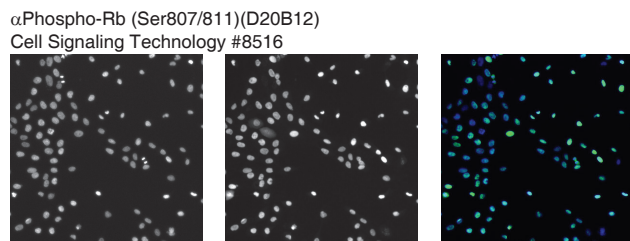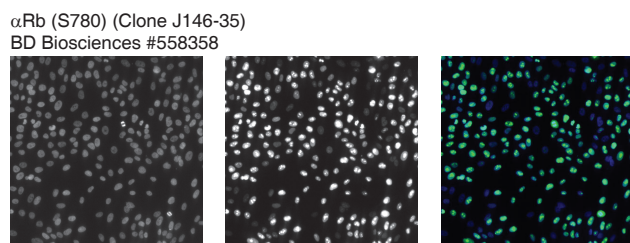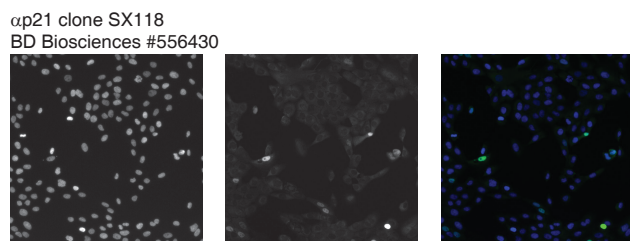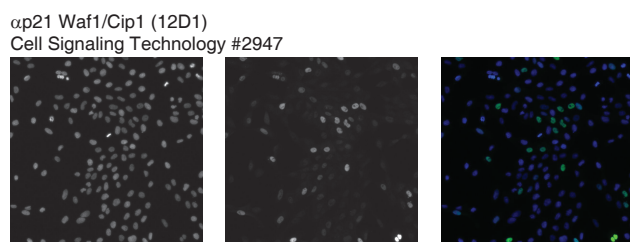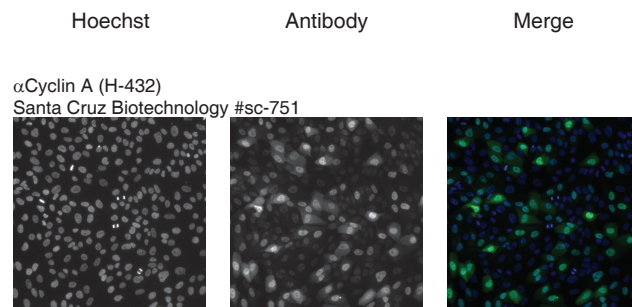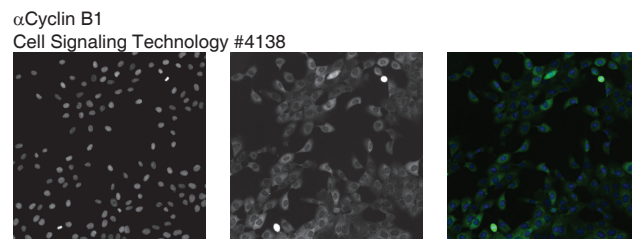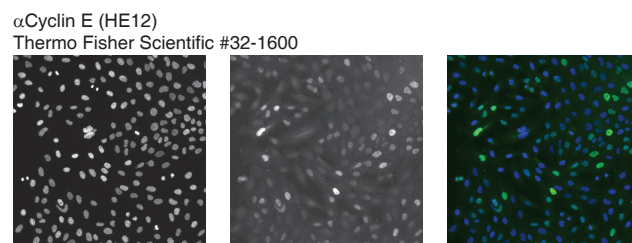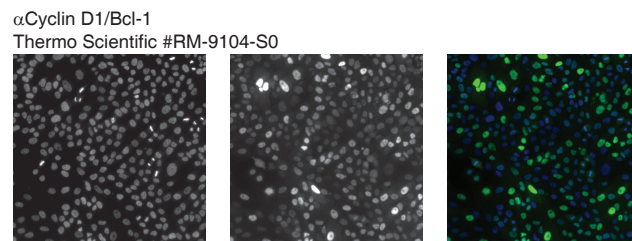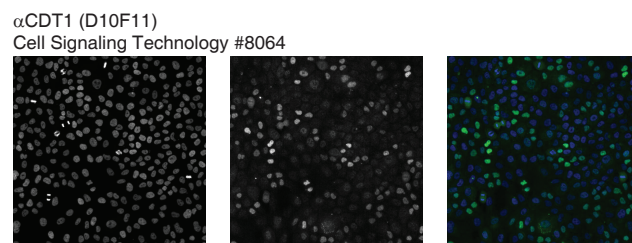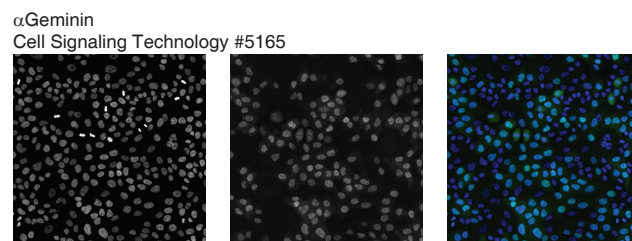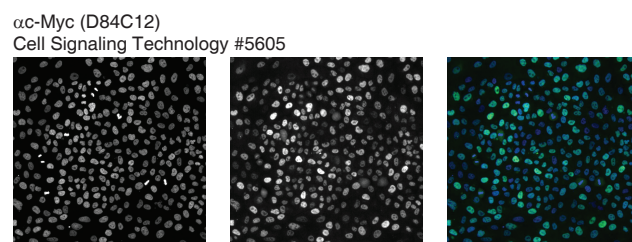

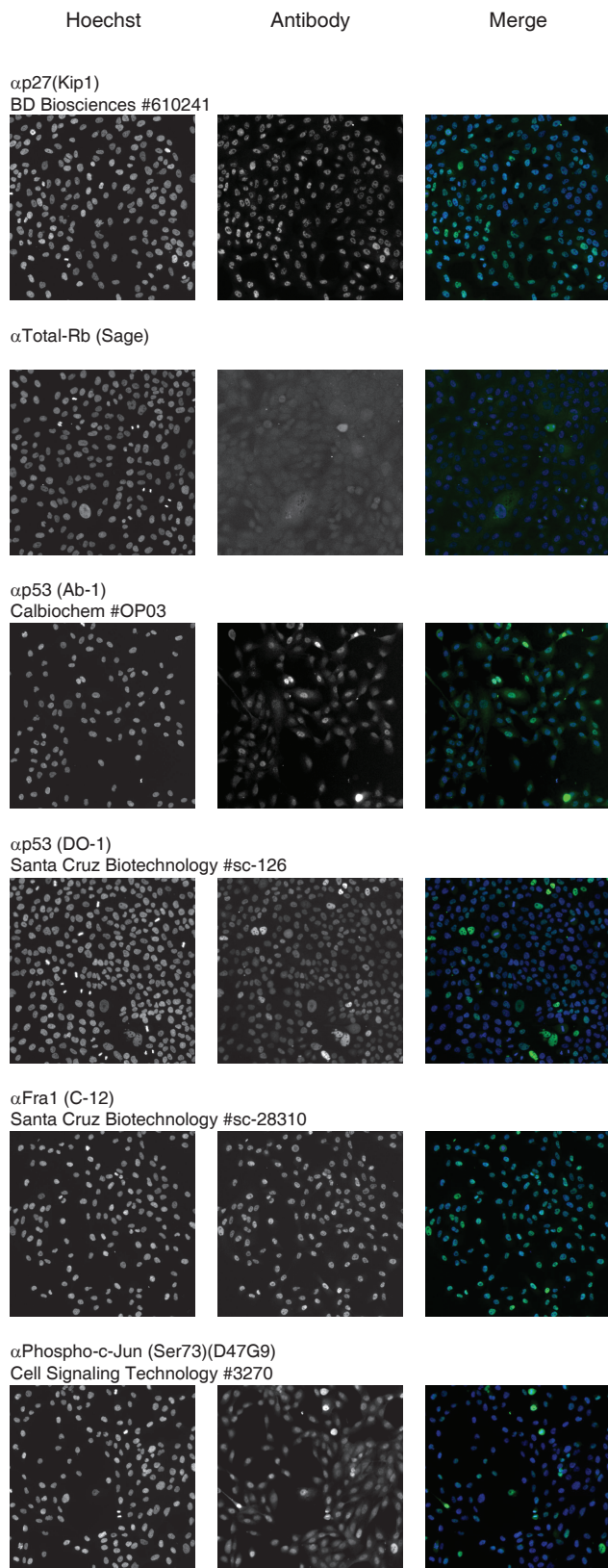

Supplement: S7 Fig — Abbreviation: IF, immunofluorescence. (PDF) [file pbio.2003268.s007.pdf]

A.

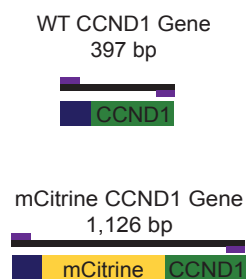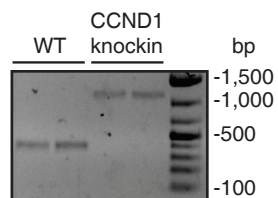

B.

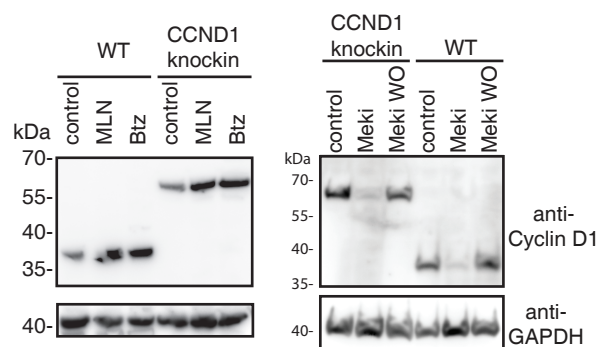

C.

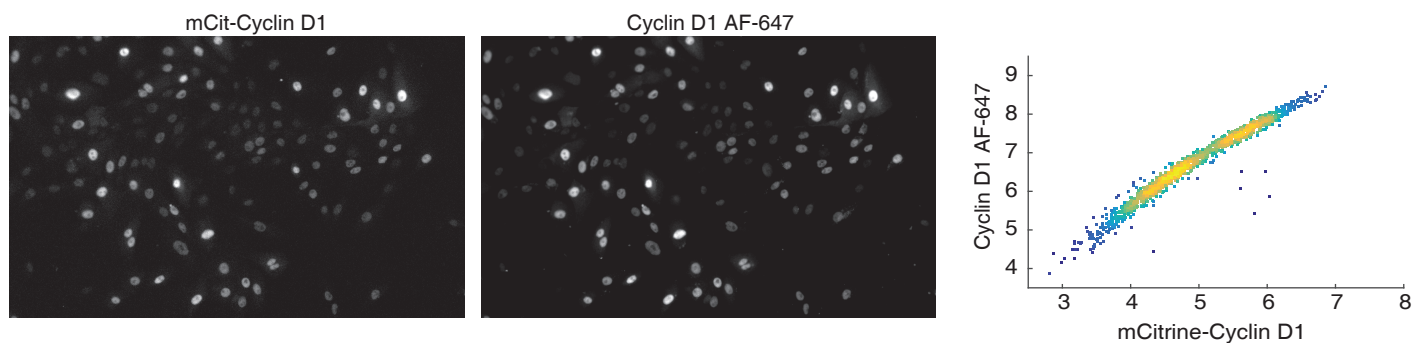

Supplement: S8 Fig — (A) Left: The expected DNA band sizes for the wild-type CCND1 gene and the mCitrine-Cyclin D1 fusion. Right: PCR amplification of the CCND1 gene in parental and mCitrine-CCND1 knock-in cells run in duplicate on 0.8% agarose gel. The mCitrine gene was knocked into both CCND1 alleles resulting in the absence of the wild-type band in the mCitrine-CCND1 knock-in cells. Bands were excised and sequenced as additional verification. (B) The mCitrine-Cyclin D1 knock-in MCF10A cell line responds in the same way as untagged Cyclin D1 in parental MCF10A cells. WT: parental MCF10A cells. CCND1 knock-in: MCF10A cells with mCitrine knocked into the CCND1 locus to produce a mCitrine-Cyclin D1 fusion protein. MLN: 1.4 μM MLN4924 treatment for 2 hours. Btz: 1 μM Bortezomib treatment for 2 hours. Meki: 100 nM PD0325901 treatment for 32 hours. Meki WO: 100 nM PD0325901 treatment for 32 hours followed by drug washout and return to full growth medium for 6 hours. (C) mCitrine intensity linearly correlates with Cyclin D1 antibody staining in the mCitrine-Cyclin D1 knock-in cell line. Left: representative images of mCitrine signal and Cyclin D1 antibody staining in the same cells. Right: Quantification of the images; axes are natural log scale. Abbreviation: WT, wild type. (PDF) [file pbio.2003268.s008.pdf]

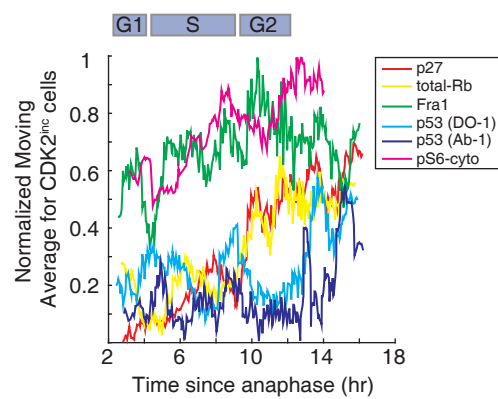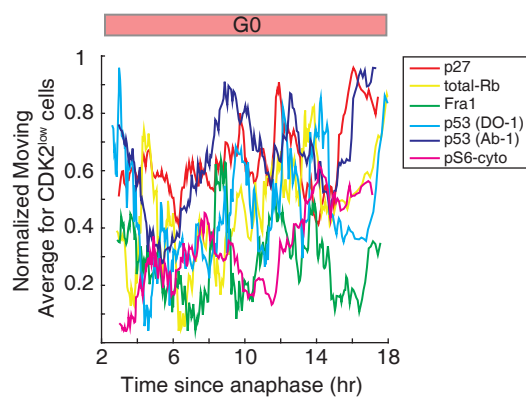

Supplement: S9 Fig — Moving average traces from S5 Fig Column 2 were normalized such that the minimum signal experienced between CDK2inc and CDK2low data for a given protein was set to 0, and the maximum signal experienced between CDK2inc and CDK2low data for this protein was set to 1. Abbreviation: CDK2, Cyclin-Dependent Kinase 2. (PDF) [file pbio.2003268.s009.pdf]
